# Target, suspect and non-target screening analysis from wastewater treatment plant effluents to drinking water using collision cross section values as additional identification criterion

**DOI:** 10.1007/s00216-021-03263-1

**Published:** 2021-03-25

**Authors:** Vanessa Hinnenkamp, Peter Balsaa, Torsten C. Schmidt

**Affiliations:** 1 IWW Water Centre, Moritzstraße 26, 45476 Muelheim an der Ruhr, Germany; 2 Instrumental Analytical Chemistry and Centre for Water and Environmental Research (ZWU), Universitaetsstrasse 5, 45141 Essen, Germany

**Keywords:** LC-IM-HRMS, Non-target screening, Micropollutants, Drinking water, CCS value

## Abstract

The anthropogenic entry of organic micropollutants into the aquatic environment leads to a potential risk for drinking water resources and the drinking water itself. Therefore, sensitive screening analysis methods are needed to monitor the raw and drinking water quality continuously. Non-target screening analysis has been shown to allow for a more comprehensive investigation of drinking water processes compared to target analysis alone. However, non-target screening is challenging due to the many features that can be detected. Thus, data processing techniques to reduce the high number of features are necessary, and prioritization techniques are important to find the features of interest for identification, as identification of unknown substances is challenging as well. In this study, a drinking water production process, where drinking water is supplied by a water reservoir, was investigated. Since the water reservoir provides surface water, which is anthropogenically influenced by wastewater treatment plant (WWTP) effluents, substances originating from WWTP effluents and reaching the drinking water were investigated, because this indicates that they cannot be removed by the drinking water production process. For this purpose, ultra-performance liquid chromatography coupled with an ion-mobility high-resolution mass spectrometer (UPLC-IM-HRMS) was used in a combined approach including target, suspect and non-target screening analysis to identify known and unknown substances. Additionally, the role of ion-mobility-derived collision cross sections (CCS) in identification is discussed. To that end, six samples (two WWTP effluent samples, a surface water sample that received the effluents, a raw water sample from a downstream water reservoir, a process sample and the drinking water) were analyzed. Positive findings for a total of 60 substances in at least one sample were obtained through quantitative screening. Sixty-five percent (15 out of 23) of the identified substances in the drinking water sample were pharmaceuticals and transformation products of pharmaceuticals. Using suspect screening, further 33 substances were tentatively identified in one or more samples, where for 19 of these substances, CCS values could be compared with CCS values from the literature, which supported the tentative identification. Eight substances were identified by reference standards. In the non-target screening, a total of ten features detected in all six samples were prioritized, whereby metoprolol acid/atenolol acid (a transformation product of the two β-blockers metoprolol and atenolol) and 1,3-benzothiazol-2-sulfonic acid (a transformation product of the vulcanization accelerator 2-mercaptobenzothiazole) were identified with reference standards. Overall, this study demonstrates the added value of a comprehensive water monitoring approach based on UPLC-IM-HRMS analysis.

**Graphical abstract:**

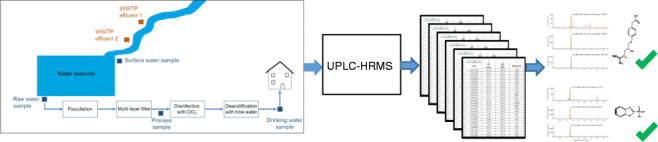

**Supplementary Information:**

The online version contains supplementary material available at 10.1007/s00216-021-03263-1.

## Introduction

In drinking water treatment processes, the removal of particles by flocculation, filtration or slow-sand filtration is commonly the first step of treatment. Optionally, advanced oxidation processes (AOP) such as ozonation, UV treatment or photocatalysis can also be applied to remove dissolved substances, and treatment trains of AOP with adsorption by activated carbon or biologically activated carbon are used as well [1–3]. An important aspect in drinking water treatment is disinfection. This is commonly accomplished using chlorine, chlorine dioxide, UV treatment or ozone [4]. However, disinfection by-products can be formed, and out of the 600–700 known disinfection by-products, some are potent cytotoxicants, genotoxicants and carcinogens [5]. Furthermore, it is reported that more than 50% of the total organically bound halogens stemming from chlorination are not identified, and for other disinfectants such as ozone or chlorine dioxide, even less is known on the occurrence of oxidation by-products [6]. The drinking water directive [7] regulates the microbiological, chemical and radiological requirements of the drinking water suppliers. For the regulation of organic compounds, with the exception of pesticides and biocides, limit values exist for only a few organic chemicals, which have to be monitored regularly. Therefore, contamination of drinking water by hazardous substances may go unnoticed. For some non-regulated compounds, health-related values are derived due to the absence of a complete toxicological assessment. Depending on the mode of action, these values range from 0.01 to 3.0 μg/L. Liquid chromatography coupled with high-resolution mass spectrometry (LC-HRMS) has the potential to detect a broad spectrum of organic substances and has been used previously for the analysis of drinking water by target, suspect or non-target screening analysis [8–12]. In target analysis, known substances are analyzed based on reference standards for identification and quantification. For suspect screening, compounds are searched for by their exact mass, derived from the known molecular formulas of the substances [13]. Furthermore, for extended security of identification, retention times, fragment ions, collision cross section (CCS) values (if determined) and isotope ratios can be used. This approach allows for the screening of a large number of compounds without the need for reference standards [14]. However, in terms of identification, substances are only considered as unequivocally identified if these are confirmed by a reference standard [15]. Otherwise, substances are considered tentatively identified. Non-target screening considers all (or only certain) signals detected in full-scan mode without prior information. For the detection of *m/z* at a given retention time with a given intensity, the term feature is frequently used [16]. One of the challenges in non-target screening analysis is the data processing, including peak detection, grouping of peaks which may belong to one compound (adducts, isotopes and in-source fragments), annotation or subtraction of blank peaks, and alignment of samples and sample replicates [16–18]. Such steps are important to reduce the complexity of the data. In order to find the most relevant features, prioritization of signals of interest is an important step, because the identification of several hundreds or even thousands of compounds in environmental samples is impossible. Various prioritization techniques can be found in the literature, including intensity-based approaches, search for a characteristic isotopic pattern, investigation of transformation products during treatment processes or the use of effect-directed analysis (EDA), where fractions (e.g., fractionated by HPLC) are subjected to toxicological tests followed by attempts to identify substances that trigger toxicological effects [19–22]. The different approaches are summarized in a review article by Hollender et al. [17]. Regarding the identification of unknowns, databases such as ChemSpider [23], PubChem [24] or MassBank [25] are useful to find potential compounds for identification by entering accurate mass (by selection of certain quasi-molecular ions). Additionally, MS/MS spectra of suspected substances can be compared with literature spectra or in silico predictions in order to restrict database matches [26, 27]. Models for the prediction of the retention time in LC-MS are reported, which can also be used for an improved identification in a suspect and/or non-target screening approach [28, 29]. However, with these approaches, fully unknown chemicals which are thus far unreported cannot be identified. In these cases, further experimental information is required using complementary techniques to MS such as nuclear magnetic resonance spectroscopy (NMR). Non-target screening in the field of water analysis described previously in the literature is based predominantly on LC-HRMS systems with electrospray ionization (ESI), using either a quadrupole time-of-flight (Q-TOF) or an Orbitrap mass analyzer [13, 16, 19, 30]. For drinking water process evaluation, Müller et al. [30] demonstrated a non-target screening approach using temporal, spatial and process-based relationships to compare different samples. More recently, Bader et al. [31] developed a classification strategy for feature signals based on observed fold changes during the drinking water treatment process.

Within the last decade, the use of ion mobility (IM) separation coupled with high-resolution mass spectrometry has gained considerable interest. The coupling of IM (separation timescales in milliseconds) with time-of-flight mass spectrometry fits well because of the high scan rates of TOF instruments (on the microsecond scale) [32]. Therefore, it is possible to separate isobaric substances which cannot be distinguished by their *m/z* [33]. Regarding identification of substances, the IM-derived CCS values can be used as further identification criteria in addition to *m/z*, retention time, isotopic pattern and fragment ions. It has been shown that CCS values are not affected by different matrices [34–36]. Thus, CCS values have a high potential to improve the confidence of compound identification [35]. Several studies have already published CCS databases for different compound classes [34–42], which can be used to compare experimental CCS values. However, especially in environmental analysis, only a few databases exist that can be used for such comparisons. Furthermore, CCS prediction methods were described in the literature, and CCS values were predicted even with median errors of ≤2% [43–45]. Another benefit of using IM-MS is the background filtering of interfering substances in MS spectra, especially in complex matrices, where co-eluting substances can influence the fragment ion spectra, which can lead to false interpretations.

The objective of this work is to identify known and unknown contaminants encountered in drinking water produced from a water reservoir. In the water reservoir, surface water is supplied which is influenced by anthropogenic emissions. Ultra-performance liquid chromatography coupled with ion mobility quadrupole time-of-flight mass spectrometry (UPLC-IM-Q-TOF-MS) was used to investigate the anthropogenic emissions containing contaminants from wastewater treatment plant (WWTP) effluents reaching the drinking water, because this indicates that these compounds cannot be removed by the drinking water production process. For this purpose, a combined approach using a quantitative screening, suspect screening and non-target screening was applied to identify known and unknown substances. Additionally, the potential of the IM-derived CCS values is discussed to support the confidence of identification of known and unknown substances.

## Materials and methods

### Chemicals

Analytical standards were purchased from Neochema (Bodenheim, Germany), LGC Standards (Wesel, Germany), Riedel-de-Haen (Seelze, Germany), Sigma-Aldrich (Taufkirchen, Germany), Syngenta (Basel, Switzerland) and BASF (Ludwigshafen, Germany). Deuterated internal standards were purchased from LGC Standards (Wesel, Germany). Methanol, acetonitrile and ultrapure water (all LC-MS grade) were purchased from Biosolve (Valkenswaard, Netherlands). Formic acid (LC-MS grade) was obtained from VWR International (Langenfeld, Germany). Standard solutions were prepared in acetonitrile and stored at 4–8 °C, and dilutions were produced in ultrapure water. A pH value of 2.5 ± 0.2 was adjusted using formic acid for the aqueous standards solutions, samples and blank (blank contains ultrapure water) measurements. Leucin-enkephalin (100 pg/μL) dissolved in acetonitrile/water (1:1) was used as lock mass and was purchased from Waters (Manchester, UK). The Vion calibration mixture (IMS/TOF calibration kit) was obtained from Waters (Manchester, UK).

### Samples

Water samples, including one drinking water, two WWTP effluents, one surface water, one raw water and one process water sample (each 100 mL), were taken on November 5, 2018, and were immediately analyzed after sampling. One-milliliter samples and blanks were spiked with 5 μL of a deuterated internal standard mixture (dissolved in acetonitrile) containing acesulfame-d4, atrazine-d5, desethyl atrazine-d6, chloramphenicol-d5, diclofenac-d4, diuron-d6, methamidophos-d6 and pendimethalin-d5 in a concentration of 2 mg/L. These internal standards were chosen due to their retention distribution over the whole chromatogram (the specific retention times can be found in Table S1 and Table S2 in the Supplementary Information [ESM]). Samples with visible suspensions were centrifuged for 15 min at 3000×*g* prior to injection. For later experiments, aliquots (1 mL) of each sample were frozen at −30 °C.

### UPLC-IM-Q-TOF-MS method

The method was previously described in brief in Hinnenkamp et al. [46] but is further explained in the following. An Acquity UPLC I-Class (Waters) coupled to a Vion IM-Q-TOF MS (Waters) was used. For the chromatographic separation, a BEH amide (2.1 × 5 mm) 1.8 μm precolumn connected with an HSS T3 (2.1 × 100 mm) 1.8 μm main column was utilized. Ultrapure water and methanol were used as mobile phase, both containing 0.1% formic acid. A flow rate of 0.35 mL/min at a column temperature of 40 °C was applied. A direct injection of a large volume of 100 μL aqueous sample was used. For gradient settings, the elution started with 100% water, holding for 1 min. Within 11.5 min, the proportion of methanol increased to 99% and was held for 2 min. Afterwards, the eluent was set to initial conditions (100% water) and held for 5 min. Electrospray ionization (ESI) was operated in ESI positive and ESI negative mode in different runs. Nitrogen was used as desolvation and cone gas. Desolvation and source temperature were set to 500 °C and 150 °C, respectively. Desolvation gas flow and cone gas flow were adjusted to 800 L/h and 50 L/h, respectively. Cone voltage was set to 20 V, and a capillary voltage of 0.8 kV was applied. A scan time of 0.3 s was adjusted. High-definition (HD) MSE acquisition mode was used, in which MSE means that data are recorded at different collision energies (low-energy spectra are recorded to obtain parent ion information using a fixed low collision energy, whereas high-energy spectra are recorded in a collision ramp for the detection of fragment ions). HDMSE means the introduction of the ion mobility separation prior to MS detection in addition to the MSE-only mode. Low-energy spectra were recorded at 4 eV, and high-energy spectra in the range of 15–40 eV. For the ion mobility separation by traveling-wave ion mobility spectrometry (TWIM-MS), the following settings were used: stopper height 40 eV; trap bias 40 V; gate height 40 V; trap wave velocity 100 m/s; trap pulse height A 20 V; trap pulse height B 5 V; IMS velocity 250 m/s; IMS pulse height 45 V; gate release 2 ms. Nitrogen was used as trap and IMS gas with flow rates of 1.6 L/min and 25 mL/min, respectively, at a pressure of about 3.3 mbar. The determination of CCS values was carried out by previous calibration using acetaminophen, caffeine, sulfaguanidine, sulfadimethoxine, L-valyl-L-tyrosyl-L-valine, verapamil, terfenadine, leucine-enkephalin, reserpine and polyalanine, with *n* = 7–13 as calibration substances. A CCS accuracy of ±2% is specified by the manufacturer. Sample and blank measurements were carried out in technical triplicates.

### Drinking water production process

**Fig. 1:**
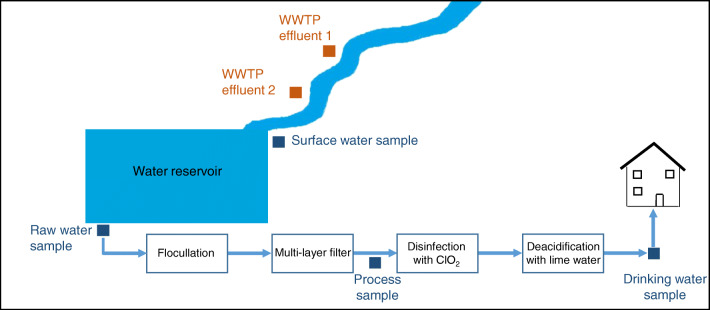
Representation of the investigated drinking water production process

During the drinking water treatment process, microorganisms and small particles are bound with flocculants, which are filtered in a multi-layer filter in the first step. In the following, disinfection with chlorine dioxide is carried out, and subsequently, deacidification by lime water is applied to bind the remaining carbonic acid. Figure 1 shows the simplified drinking water treatment process, including the sampling points studied in this work.

Upstream of the water reservoir, effluents of two WWTPs are discharged into the surface water. In the context of this study, two WWTP effluents and the surface water immediately before reaching the water reservoir were investigated in order to detect anthropogenic influences, which one may also find in drinking water. Furthermore, the raw water of the water reservoir was examined and a process water sample, taken before the addition of chlorine dioxide, and the final drinking water were analyzed.

### Data processing

For data processing, the Unifi 1.9.4 software (Waters) was primarily used. An overview of the software used for each processing step for the non-target screening can be found in Fig. S1 (see ESM).

### Quantitative screening

For the quantitative screening, an external calibration (measuring standards dissolved in ultrapure water with the concentrations: 100 ng/L, 250 ng/L, 500 ng/L, 750 ng/L and 1000 ng/L) was used to determine 143 organic micropollutants in aqueous samples. In this case, matrix effects, which occur in ESI and can influence the results, are not taken into account. Accordingly, only concentration ranges are reported. The concentration ranges are divided into the three classes: (1) < 100 ng/L, (2) between 100 and 1000 ng/L and (3) > 1000 ng/L. In cases in which no signal was detected or could not be clearly identified, the indication not detectable (n.d.) is reported. The method was validated, and limits of quantification of less or equal than 100 ng/L were achieved. For more details, see Hinnenkamp et al. [47].

### Suspect screening

For suspect screening, a total of three scientific libraries were separately processed in Unifi. Identification was based on the confidence levels published by Celma et al. [35], but retention time indices were not considered, and for the fragment spectrum match, only one fragment had to match. The first processed database was adapted from Wode et al. [48]. This database contains 1125 entries on water-relevant substances, including pharmaceuticals, pesticides and other relevant substances such as industrial chemicals. It comprises the corresponding molecular formulas, adducts and fragment ions for ESI+ data. Note that in this study and in our previous work [47], mass errors are given in mDa instead of the frequently used relative value in ppm, because the relative mass errors of smaller molecules compared to larger molecules are very large (e.g., *m/z* of 100.0000 and 1000.0000 with mass errors of 2 mDa provide relative mass errors of 20 ppm and 2 ppm, respectively). If high relative mass errors are allowed, the proportion of false positive results increases, but on the other hand, relative mass errors that are too low can result in false negative results. Hence, the tentative identification was based on the accurate mass (± 2 mDa), the isotopic ratio (≤ 30% error) and the agreement of at least one fragment ion (± 2 mDa). The second processed database was the CCS pesticides database from Waters. Included in the pesticide database are 608 entries for molecular formulas, adducts, CCS values and fragment ions for ESI+ data. The tentative identification was carried out via accurate mass (± 2 mDa), isotope ratio (≤ 30% error), the agreement of at least one fragment ion (± 2 mDa) and additionally the CCS value (deviation maximum 2%). The third database used was an in-house database and contains 90 entries for substances beside the 143 target substances considered in the quantitative screening. These substances were previously measured with the existing method in ESI+ and ESI– mode (molecular formula, the found quasi-molecular ion, retention time, CCS value and fragment ions are entered). For a match with this database, a retention time error of ±0.05 min, mass error of ±2 mDa, CCS error of ±2% and isotope ratio error of ≤30% were allowed. Additionally, one fragment ion (± 2 mDa) had to match. In order to exclude false positive identification, all matches with the database had to occur in all triplicates, and none of the matches was present in the blank, which was checked manually.

### Non-target screening

For peak finding, a threshold value of 200 counts was set, and a retention time range from 1.5 to 14 min was selected. For the detection of *m/z* at a retention time with a CCS and response value, the term feature is used in this study. The multiple adduct finder was used in order to find [M + H]^+^, [M + Na]^+^, [M + NH_4_]^+^, [M + K]^+^ and [M + Li]^+^ in ESI+ mode and [M-H]^−^, [M + Cl]^−^, [M + HCOO]^−^ in ESI– mode. Multiple ions from one component were removed from the feature list, and only the most intense ion remained in the feature list. Note that in-source fragmentation (e.g., loss of water) was not considered. The 4D isotope clustering algorithm was applied, where isotopes from one component are grouped together during peak detection. Already identified components by quantitative screening or suspect screening and tentatively identified compounds by suspect screening were also removed from the feature lists.

In the next step, a script programmed in Origin 2018b [49] was used to merge triplicates from each sample, where only features occurring in all three sample feature lists and three blank sample feature lists were further considered. Using this script, feature lists of the three replicates of each sample were compared. The features are defined by their *m/z*, retention time, CCS value and response value. It is important to set tolerance values for these to determine which features can be merged from different measurements and thus different feature lists. These tolerance values were set to ±2 mDa for the *m/z*, ± 0.05 min for the retention time and ± 2% for the CCS value, and for the response value a maximum relative standard deviation of 30% was selected. Features exceeding at least one tolerance value were removed from the list. After that, features contained in the blank sample were subtracted. The merging of triplicates and the blank reduction was controlled by internal standards, which have to be recovered in the lists of merged triplicates, and on the other hand, they have to be removed in the blank reduced list. Atrazine-d5, desethyl atrazine-d6, diclofenac-d4, diuron-d6, methamidophos-d6 and pendimethalin-d5 were used for ESI+ data, and acesulfame-d4, chloramphenicol-d5, diclofenac-d4 and diuron-d6 were used for ESI– data. Detailed information can be found in Tables S1 and S2 (see ESM).

In a next step, feature intersections of the two WWTP effluents, the surface water, raw water, process water and the final drinking water sample were determined, and only features occurring in all feature lists of all samples were further considered to examine WWTP effluent-derived organic substances reaching drinking water. The resulting features were confirmed by manual checking of the peak form (to avoid false positive peak findings) and plausible intensity distribution over all samples (to avoid peaks which have similar response values over all samples and which may indicate sample contamination). All remaining features were subjected to a molecular formula finder using the elucidation tool set. The elements carbon, hydrogen, nitrogen, oxygen, sulfur, phosphor, fluorine, chlorine and bromine were selected, and a maximum mass error of ±1 mDa for the molecular formula was adjusted. Molecular formulas with an i-fit confidence ≥80% were further considered, where i-fit confidence means a score of each formula based on the theoretical isotope ratio, number of double bonds and further chemical rules (carbon/hydrogen ratio, carbon/heteroatom ratio and Senior rule). The assigned molecular formulas were entered in the open-source database FOR-IDENT [50]. Features with matches in the database were prioritized, and mass spectra were compared as far as possible, or in silico fragmentation was applied for increasing the identification confidence. Therefore, mol-files downloaded from ChemSpider [23] were used and uploaded in Unifi. For a fragment ion match, a maximum mass error of ±2 mDa was set. By using the categorization of identification confidence from Celma et al. [35], features with a matching fragment spectrum were tentatively identified (level 2) but without considering retention time indices and as far as possible reference standards for level 1 or disproving identification were purchased. A graphical flowchart of the data treatment for non-target screening analysis is in Fig. S1 (see ESM). Note that parts of the non-target screening method were published in Hinnenkamp et al. [46].

## Results and discussion

### Quantitative screening

Results of the quantitative screening of 143 micropollutants are summarized in ESM Table S3. Out of these, 60 substances were found and quantified in at least one sample. The resulting concentration ranges are summarized in Table 1.

**Table 1:**
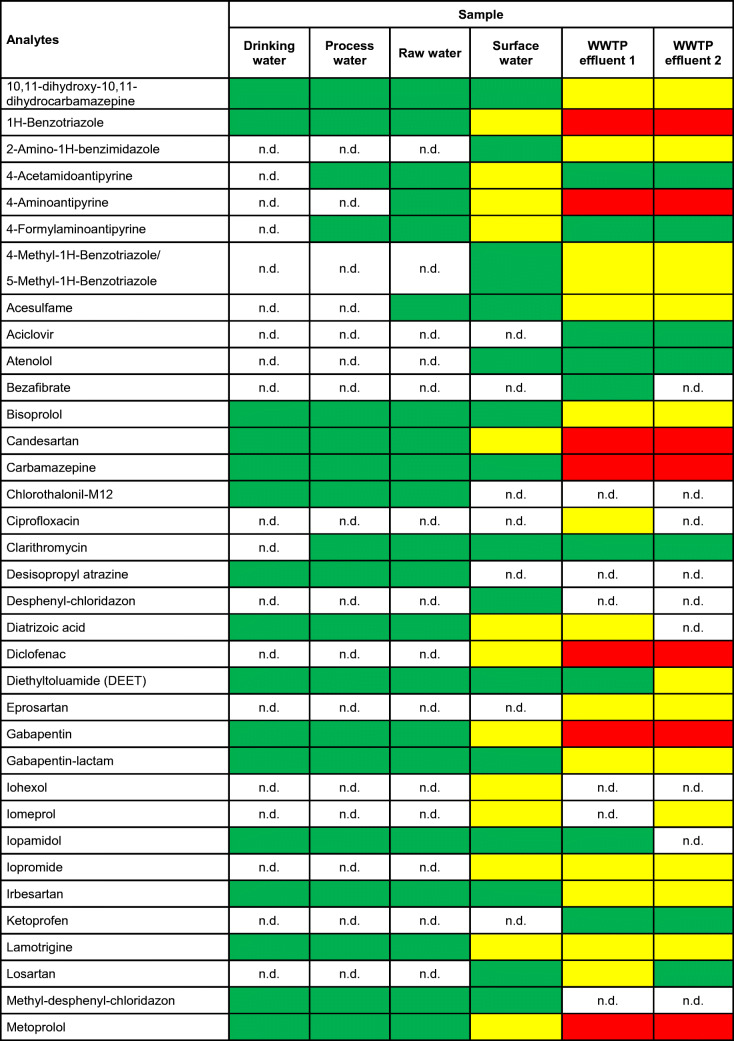

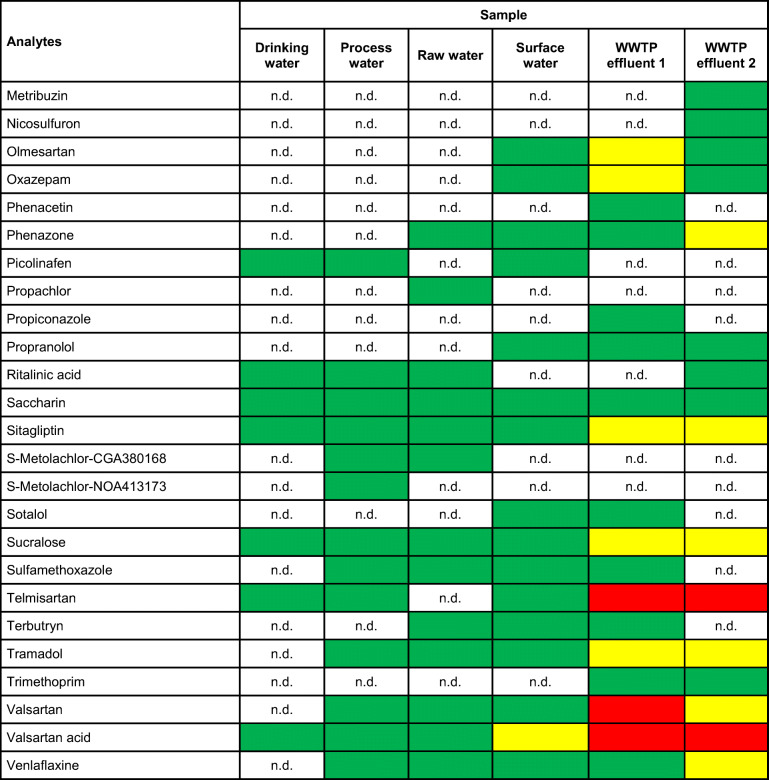
Positive findings from the quantitative screening of the six investigated samples. Green marked fields indicate the concentration range < 100 ng/L, yellow marked fields indicate the concentration range between 100 and 1000 ng/L and red marked fields indicate the concentration range > 1000 ng/L. For compounds not detected, n.d. is indicated

This investigation showed that for the WWTP effluent samples, all concentration ranges are present, whereas in the surface water sample, only the concentration ranges <100 ng/L and 100–1000 ng/L were found. For the raw, process and drinking water sample, only the concentration range < 100 ng/L was determined the in case of positive findings. Overall, 15 target compounds were detected in all samples.

### Suspect screening

For the suspect screening, a total of 42 compounds matched with the specified criteria in one or more samples. Eight of these (flecainide, amisulpride, clindamycin, fexofenadine, melamine, sulpiride, verapamil and anhydroerythromycin) could be confirmed by reference standards comparing the accurate mass (± 2 mDa), retention time (± 0.05 min), CCS value (± 2%) and the fragment ion spectrum. In the drinking water sample, only flecainide, which is used as an antiarrhythmic drug, matched and could be identified. The remaining 34 compounds were tentatively identified. All results from the suspect screening are listed in Table S4 (see ESM). CCS values determined for the identified and tentatively identified substances were compared to literature values (ESM, Table S5). For this purpose, the PubChem database was used [24]. CCS values determined with different ion mobility instrument types (e.g., drift tube ion mobility mass spectrometry and traveling-wave ion mobility mass spectrometry) cannot be used without care indepently from the instrument type [51], and it must be noted that CCS values determined by  TWIM-MS are derived from a CCS calibration, and the calibration in TWIM-MS is strongly dependent on the compounds used [37]. Therefore, only CCS values which were also determined with TWIM-MS and with the same calibration compounds were used as reference values in the comparison. From the total of eight identified compounds, CCS values of six could be compared to literature CCS values and showed deviations < ± 2% for all CCS values (comparing CCS values from samples with the literature CCS values), with a median deviation of 0.25%. Furthermore, CCS values from the literature were compared with the CCS values of the reference standards, and deviations of < ± 2% with a median deviation of 0.71% were calculated. Regarding the total of 34 tentatively identified substances, CCS values for 19 could be compared with one or more literature CCS values, resulting in a median deviation for all CCS values of 0.51%. For 17 compounds, a CCS deviation of < ±2% was achieved. For the β-blocker alprenolol, a CCS deviation of 4.4% was calculated, and due to this large deviation, alprenolol was removed from the list of tentatively identified substances. In the case of the antibiotic nalidixic acid, a deviation of 2.7% from a CCS value obtained from Celma et al. [35] was calculated. Considering a tolerance value of ±2%, nalidixic acid would no longer be present in the list of tentatively identified substances. On the other hand, with the CCS value obtained from Tejada-Casado et al. [52], a smaller deviation of 1.2% was calculated, which would fit with the accepted tolerance value of <2%. In the end, nalidixic acid was kept as a tentatively identified compound, but this result clearly shows that despite the above mentioned selection criteria, currently different CCS value sources may lead to different results in suspect screening.

### Non-target screening

Feature lists for all samples containing *m/z*, retention time, CCS values and response values of each feature were transferred to Origin 2018b for processing. The two data reduction steps (merging of triplicates and blank reduction) were validated by the spiked internal standards for quality assurance. As expected, these were found after pooling the triplicates in the feature lists and were completely removed after blank reduction. Table 2 shows the results for each processing step considering the drinking water sample.

**Table 2:** Overview of the outcome of the data reduction procedure for the drinking water sample in ESI+ and ESI– mode. The full description of data processing can be found in the materials and methods section

Processing step	Remaining number of features in ESI+ mode	Remaining number of features in ESI– mode
Drinking water sample (first replicate)	2280	771
Multiple ion correction	2215	769
Features in all sample triplicates	1147	269
Blank reduction	409	134
Formation of intersections (WWTP effluent samples, surface water, raw water, process water and drinking water sample)	52	20
Manual checking of the peaks	25	11
Proposed molecular formula with an i-fit confidence ≥80%	7	3

The first step of data reduction, meaning the multiple ion correction, removed 3% (ESI+ mode) and 0.3% (ESI– mode) of the data. On the other hand, the merging of triplicates removed a noticeably higher fraction of 48% (ESI+ mode) and 65% (ESI– mode) of the multiple-ion corrected data. By the blank reduction step, a high fraction of features (64% in ESI+ mode and 50% in ESI– mode) were further removed as well. Overall, from a total of 2280 and 771 features, only seven and three features remained in ESI+ and ESI– mode, respectively, which were prioritized for further identification (Table 3). The response values detected in the samples are depicted in Figs. S2 for ESI+ and S3 for ESI– (see ESM).

**Table 3:** Remaining features after data reduction (*m/z*, RT and CCS were averaged over all samples)

Prioritized features	ESI mode	*m/z*	Retention time [min]	CCS [Å^2^]	Molecular formula for [M + H]^+^	Molecular formula for [M – H]^−^	i-fit confidence [%]	Number of matches in the FOR-IDENT database
Feature 1	+	289.0531	4.59	153.5	C13H6F2N4O2	–	100	–
Feature 2	+	291.1416	5.26	154.9	C11H21F3O5*	–	93	–
Feature 3	+	335.1679	5.79	164.8	C13H25F3O6*	–	96	–
Feature 4	+	268.1545	6.06	168.3	C14H21NO4	–	88	2
Feature 5	+	423.2203	6.60	184.0	C17H33F3O8*	–	90	–
Feature 6	+	174.1852	5.97	145.4	C10H23NO	–	100	2
Feature 7	+	248.2229	6.00	163.2	C13H29NO3	–	85	–
Feature 8	–	213.9643	6.21	138.5	–	C7H5NO3S2	100	1
Feature 9	–	297.0809	8.23	177.0	–	C12H22F2P2S	99	–
Feature 10	–	301.0396	6.07	156.3	–	C12H14O7S	94	–

*These features rather indicate an [M + Na]^+^ adduct (as described in the text), thus the given molecular formula is likely incorrect

**Fig. 2:**
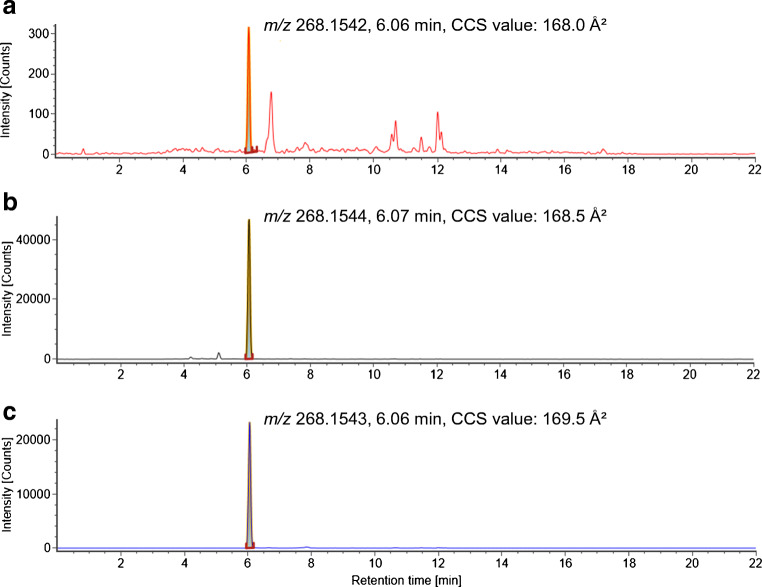
Extracted ion chromatograms of metoprolol/atenolol acid for **a**) the drinking water sample, **b**) the WWTP effluent 2 sample and **c**) for the reference standard (500 ng/L) in a 30-ppm mass window

**Fig. 3:**
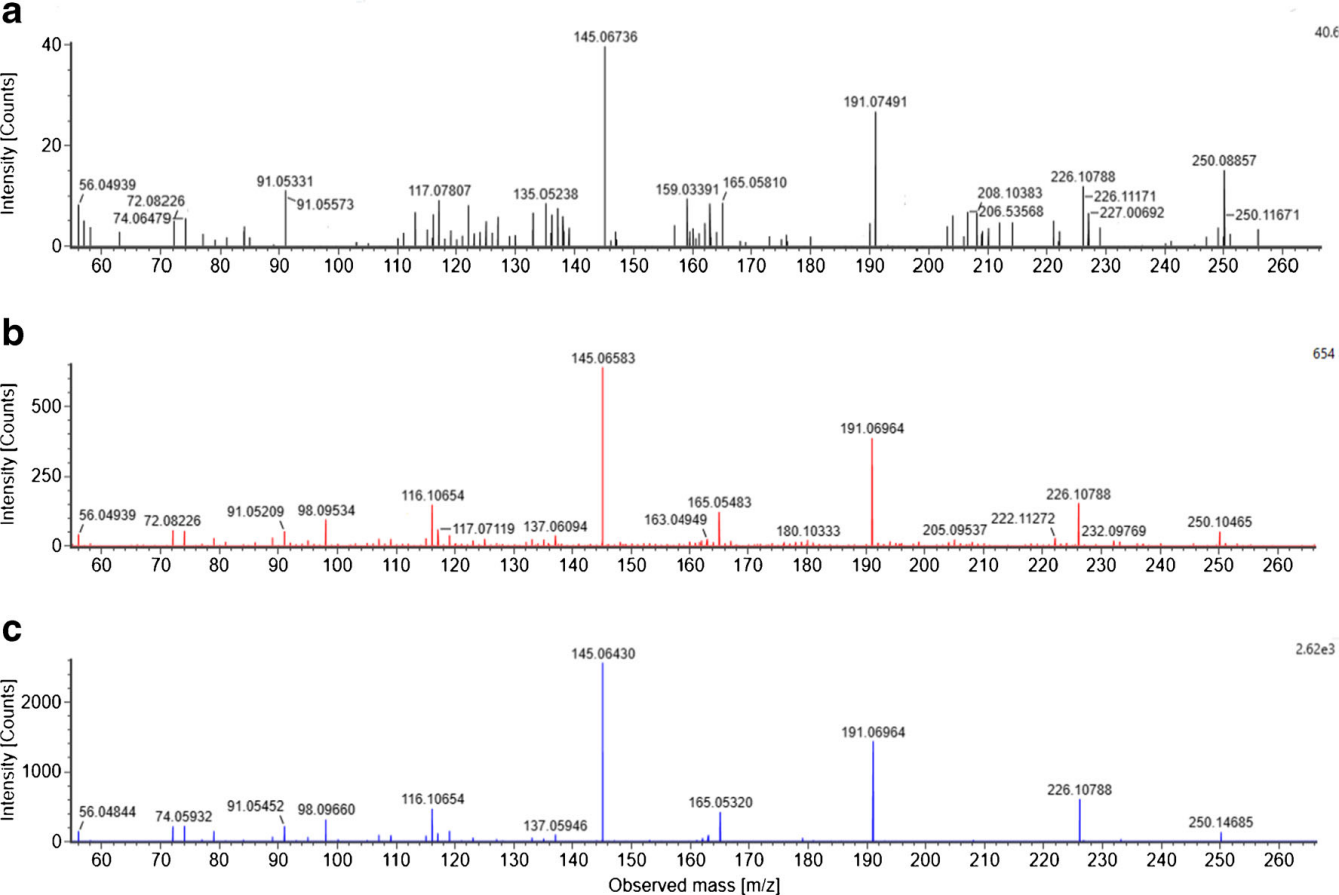
Fragment ion spectra for **a**) the drinking water sample, **b**) the WWTP effluent 2 sample and **c**) for the reference standard (500 ng/L). Spectra were recorded by a collision energy ramp from 15 eV to 40 eV from the precursor *m/z* 268.1545 using the HDMSE scan mode

Regarding the ESI+ data, for one of these features (feature 4) with *m/z* 268.1545, retention time of 6.06 min and a CCS value of 168.3 Å^2^, a molecular formula of C_14_H_21_NO_4_ was proposed and matched with the FOR-IDENT database for metoprolol acid/atenolol acid (ESM, Fig. S4), a transformation product of both metoprolol and atenolol that are used as beta blockers. The occurrence of metoprolol acid/atenolol acid in wastewater-impacted surface water has previously been reported [53]. Another match for the same feature was obtained for the fungicide diethofencarb. However, diethofencarb use is not authorized in Germany, making the plausibility of a positive result less likely, and was not further investigated. A reference spectrum for metoprolol acid/atenolol acid was received by PubChem (ESM, Fig. S5) and matched with the measured fragments (WWTP effluent 2 sample) of *m/z* 116.1065, 145.0658, 165.0548, 191.0696 and 226.1079 with mass differences below 2 mDa. Only the fragment ion *m/z* 250.1047 showed a higher deviation of 38.4 mDa in comparison to the reference fragment ion. However, this fragment ion was detected with a very low intensity. For metoprolol acid/atenolol acid, experimental CCS values were not found in the literature. Therefore, the CCS prediction tool CCSbase [54] developed by Ross et al. [55] was used. They reported that for 94% of a test set containing more than 600 CCS values, a prediction error of ≤5% was achieved. In the CCSbase web interface, the SMILES code for metoprolol acid/atenolol acid (obtained from PubChem) was entered, and a CCS value of 162.7 Å^2^ for the [M + H]^+^ ion was predicted, which differs by 3.3% from the CCS value measured for that feature. A reference standard of metoprolol acid/atenolol acid was purchased and compared to sample measurement results by *m/z*, retention time, CCS value (Fig. 2) and fragment ion spectra (Fig. 3).

The comparison of the drinking water sample, WWTP effluent 2 sample and the reference standard indicates the match in *m/z* value (deviation <2 mDa), retention time (deviation <0.05 min) and CCS value (deviation <2%). The high-intensity fragment ions in the WWTP effluent 2 sample (*m/z* 116.1065, 145.0658, 165.0548, 191.0696 and 226.1079) have mass differences below 2 mDa in comparison to the fragment ions in the reference spectra. Mass differences for the low-intensity fragment ions (*m/z* 56.0494, 91.0521, 98.0953 and 137.0609) were between 1.0 and 2.4 mDa. For the fragment ion with *m/z* 250.1047, the highest mass difference of 42.2 mDa was calculated. Considering the signal intensities, the distribution is similar in the samples to the reference. Overall, this confirms the presence of metoprolol acid/atenolol acid in the samples [46].

Features 2, 3 and 5 revealed an association due to their mass differences. Feature 3 is separated by 44 mass units from feature 2, and feature 5 is separated by 88 mass units from feature 3. As a further result, similar molecular formulas were obtained, assuming an [M + H]^+^ adduct. This leads to the assumption that these features belong to a homologous series. Such homologous series detected in water sample measurements by LC-HRMS have been published for other series [56–58]. For example, Verkh et al. [57] revealed homologous series for the (–CH_2_–) and (–C_2_H_2_O–) series in wastewater treatment samples using Kendrick mass defect plots [59]. Thurman et al. [56] identified two series of ethylene oxide surfactants in hydraulic fracturing flowback and produced water by a modified version of the Kendrick mass scale. The mass difference (from feature 3 to feature 2) of 44.0263 corresponds to an ethoxylated structure of (–CH_2_–CH_2_–O–) (exact mass of 44.0262), which was found also by Thurman et al. However, comparing the exact masses found in the two homologous series with the masses found by Thurman et al., which could be identified as polyethylene glycols and linear alkyl ethoxylates, did not match with the detected *m/z* values in this work, even if [M + Na]^+^ and [M + NH_4_]^+^ adducts beside [M + H]^+^ adducts were considered. Recently, Mairinger et al. [58] investigated synthetic water-soluble polymeric substances in WWTPs by suspect and non-target screening. Comparing their suspect list and the homologous series found by non-target screening with the *m/z* found in this study, these *m/z* which can be associated with a (–CH_2_–CH_2_–O–) structure could not be found. In a reprocessing step, it was checked whether further features belonging to the homologous series were detected, but were not included in the final feature list due to the high requirements by data treatment processing. Therefore, a further feature search was done in the mass range 115.0369–995.5609. During this reprocessing step, a lower threshold value of 10 counts was applied instead of 200 counts during the first data processing step.

**Fig. 4:**
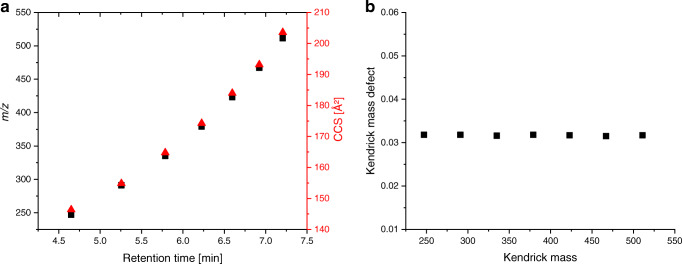
**a**) Plotting of the retention time against *m/z* as black squares and CCS values as red triangles (all adapted from the WWTP effluent 2 sample) of the homologous series features and **b**) plotting of the Kendrick mass against the calculated Kendrick mass defect

By reprocessing, four additional features (with *m/z* of 247.1153, 379.1940, 467.2468 and 511.2727) were found, which apparently belong to the same homologous series (Fig. 4a). Additionally, a Kendrick mass defect plot was constructed (Fig. 4b) and showed for these features the same Kendrick mass defect of 0.0317 ± 0.0001, which confirms the suggestion of a homologous series. The calculation of the Kendrick mass defect was based on the study of Thurman et al. where Kendrick mass defects were calculated for (–C_2_H_4_O–) structures as well (details in ESM, Table S6). Response values detected in the further samples are depicted in Fig. S6 (see ESM). Fragment ions were not detected in the high-energy spectra of all samples and all features. Further measurements of the WWTP effluent 2 sample recorded with a higher collision energy (up to 80 eV) resulted in no fragment ions as well. Because this is more often observed for sodium adducts, molecular formulas were calculated for [M + Na]^+^ adducts as well (ESM, Table S7). This indicates that for the features with *m/z* 291.1416, 335.1679, 379.1940 and 423.2203, the molecular formulas C_11_H_24_O_7_ (i-fit confidence of 70%), C_13_H_28_O_8_ (i-fit confidence of 100%), C_15_H_32_O_9_ (i-fit confidence 50%) and C_17_H_36_O_10_ (i-fit confidence 31%), respectively, can be calculated. For the other associated features, no fitting molecular formulas were determined in Unifi, but theoretically resulted in C_9_H_20_O_6_ for the feature with *m/z* 247.1153, C_19_H_40_O_11_ for the feature with *m/z* 467.2468 and C_21_H_44_O_12_ for the feature with *m/z* 511.2727. The other calculated molecular formulas were not further considered because either no association was noticeable within the homologous series for more than three features or the i-fit confidence was very low. Exemplarily, for *m/z* 335.1679 (C_13_H_28_O_8_), structures were searched in the ChemSpider database. Five results were found (ESM, Fig. S7). However, a chemical structure search in Scifinder [60] revealed that no publication dealing with the occurrence in the environment for one or more of these compounds is available. Finally, the identification confidence for these features remains at level 5.

For the other prioritized features of the ESI+ data, molecular formulas for feature 1 (*m/z* 289.0531 at 4.59 min with a CCS value of 153.5 Å^2^), feature 6 (*m/z* 174.1852 at 5.97 min with a CCS value of 145.4 Å^2^) and feature 7 (*m/z* 248.2229 at 6.00 min with a CCS value of 163.2 Å^2^) were calculated, resulting in C_13_H_6_F_2_N_4_O_2_, C_10_H_23_NO and C_13_H_29_NO_3_, respectively. Only for feature 6, matches for *N*,*N*-dibutylethanolamine and *N*,*N*-dimethyloctylamine-*N*-oxide (ESM, Fig. S8) were received with the FOR-IDENT database. An in silico fragmentation was performed in Unifi for a comparison with the measured fragment ion spectra for the WWTP effluent 1, and the results are shown in Fig. S9 (see ESM). In both cases, only two fragment ions with *m/z* 100.1124 and 118.1231 matched with the computational fragmentation, which does not support the identification of the feature as one of the two database matches. It should be noted that the in silico fragmentation does not consider rearrangements. Overall, feature 6 could not be tentatively identified as one of the matches with the FOR-IDENT database.

In ESI– mode, for two features (*m/z* 297.0809 and 301.0396), no matches with the FOR-IDENT database were found, and these were not further considered. For the remaining feature with *m/z* 213.9643 at 6.21 min with a CCS value of 138.5 Å^2^, a molecular formula match for C_7_H_5_NO_3_S_2_ with an i-fit confidence of 100% was found. In the FOR-IDENT database, a match with 1,3-benzothiazole-2-sulfonic acid (ESM, Fig. S10) was received. 1,3-Benzothiazole-2-sulfonic acid is a known transformation product of the vulcanization accelerator 2-mercaptobenzothiazole, and its occurrence in waste and surface water has been reported [61]. In ESI+ measurements, a corresponding signal for 1,3-benzothiazole-2-sulfonic acid was not detected. Schymanski et al. [20] identified 1,3-benzothiazole-2-sulfonic acid during a non-target and suspected screening for sulfur-containing surfactants in wastewater samples. They reported signals in the fragment ion spectra corresponding to a loss of SO_2_ and SO_3_ from the [M–H]^−^ ion of 1,3-benzothiazole-2-sulfonic acid. A fragment ion spectrum was obtained from PubChem (ESM, Fig. S11) that also indicates the fragments reported by Schymanski et al. Since no CCS value was available in the literature for 1,3-benzothiazole-2-sulfonic acid, a CCS value predicted by CCSbase was used and revealed a CCS value of 141.2 Å^2^, which differs by −1.9% from the measured CCS value of the feature. Confirmation by a reference standard was hampered by a long delivery time of the commercially available substance, therefore, the obtained reference substance of 1,3-benzothiazole-2-sulfonic acid was measured together with a re-measurement of the drinking water sample and WWTP effluent 2 sample (which were frozen at −30 °C) in order to counteract large deviations over time, especially in the retention times. Figure S12 (see ESM) indicates the resulting extracted-ion chromatograms (EICs), and Fig. S13 (see ESM) demonstrates the fragment ion spectra. The results showed that the *m/z* values differ by less than 1 mDa. The retention time and CCS value deviations are within a permissible range of ±0.05 min for the retention time and ± 2% of the CCS value. The fragment ion spectra showed that, similar to the results by Schymanski et al., fragment ions, which suggest the loss of SO_2_ (theoretical *m/z* of 150.0019) and SO_3_ (theoretical *m/z* of 134.0070), were recorded. In the fragment ion spectrum obtained for the drinking water sample, only the fragment ion with *m/z* 134.0075 was recorded. In the fragment ion spectrum from the WWTP effluent 2 sample, which has a higher intensity in comparison to the drinking water sample, *m/z* 134.0071 and 150.0025 were recorded and matched with mass differences below 1 mDa compared to the theoretically determined fragment ions. In comparison to the reference spectra, the intensity distribution of the fragment ions showed good agreement as well. Therefore, feature 1 could be unequivocally identified as 1,3-benzothiazole-2-sulfonic acid. Furthermore, a suspected search was performed in all samples in both ESI modes for the parent compound 2-mercaptobenzothiazole (C_7_H_5_NS_2_) but was negative in all samples.

Persistent and mobile organic compounds (PMOC) as water contaminants are increasingly in the focus of environmental monitoring, because they have a high potential to pass through wastewater treatment plants and drinking water treatment processes and because of difficulties in their analysis. Reemtsma et al. [62] showed examples of PMOCs spanning a log D range at pH 7.4 of between −1 and −8. Of the two substances identified by non-target screening analysis, 1,3-benzothiazole-2-sulfonic acid with a reported log D value of −3.0 at pH 7.4 [63] clearly falls in that range, and thus may be considered as a PMOC. In contrast, metoprolol acid/atenolol acid, with a log D value of −0.44 at pH 7.5 [64], while still being rather polar is at the upper end of the PMOC range. This classification is based on polarity only, since in both cases, nothing is yet known on the persistence of the compounds. However, their detection in drinking water at least suggests sufficiently long lifetimes to be found as drinking water contaminants.

## Conclusion

A combined approach including a target, suspect and non-target screening analysis was applied for the investigation of a drinking water production process. With this strategy, an extensive analysis with only one LC-HRMS data set can be performed.

In total, 51 substances were identified by a quantitative screening of the WWTP effluents, and 19 of those were also detected in the drinking water sample. Regarding the potential human health risk from the occurrence of the detected substances in the drinking water, no limit values yet exist for the detected compounds. However, for ten compounds, health-related values are available (0.3 μg/L for 10,11-dihydroxy-10,11-dihydrocarbamazepine, candesartan, carbamazepine and valsartan acid, 1 μg/L for gabapentin, gabapentin-lactam and iopamidol, and 3 μg/L for 1H-benzotriazole, chlorothalonil M-12 and methyl-desphenyl-chloridazon). All these values are above the estimated concentration range from quantitative screening in the drinking water sample, thus no human health risk is perceived from the detected compounds.

CCS values were used for identification of targeted and suspected compounds. Especially in suspect screening, the CCS value can improve the confidence of tentative identification. Therefore, CCS databases should be extended and made publicly available. CCS prediction can also support identification, but should be used with caution, as higher deviations do not necessarily mean that it is not the predicted substance. Besides the identified and tentatively identified substances, unknown substances could be characterized, which should be included in future monitoring campaigns (even without identification) in order to survey trends in their occurrence over time.

## Supplementary Information

ESM 1 (PDF 707 kb)
