# Supplementary material for: Target, suspect and non-target screening analysis from wastewater treatment plant effluents to drinking water using collision cross section values as additional identification criterion
Source: Anal Bioanal Chem. 2021 Mar 25;414(1):425–38. doi: 10.1007/s00216-021-03263-1 (PMC8748347; doi:10.1007/s00216-021-03263-1)
Supplement: Supplementary file 1 — (PDF 707 kb) [file 216_2021_3263_MOESM1_ESM.pdf]

Supplementary Information

Target, suspect and non-target screening analysis from wastewater treatment plant effluents to drinking water using collision cross section values as additional identification criterion

Vanessa Hinnenkamp[1], Peter Balsaa[1], Torsten C. Schmidt[1,2*]

[1] IWW Water Centre, Moritzstraße 26, 45476 Muelheim an der Ruhr, Germany

[2] Instrumental Analytical Chemistry and Centre for Water and Environmental Research (ZWU), Universitaetsstrasse 5, 45141 Essen, Germany

*Corresponding Author: Torsten C. Schmidt, Universitaetsstr. 5, 45141 Essen, Germany, e-

mail torsten.schmidt@uni-due.de, ORCID: 0000-0003-1107-4403

Additional tables and figures

Table S1. LC-HRMS data for the internal standards used for ESI+ mode measurements.

Substance	RT [min]	Adduct	CCS [Å²]	Fragment ion 1	Fragment ion 2	Fragment ion 3	Fragment ion 4
Atrazine-d5	10.14	[M+H]+	151.9	179.0857	147.0293	137.0639	101.0873
Desethyl atrazine-d6	7.89	[M+H]+	142.4	147.0292	111.0525	105.0074	
Diclofenac-d4	11.76	[M+H]+	158.7	218.0671	254.0435	183.0982	
Diuron-d6	10.26	[M+H]+	150.2	187.9668	159.9719	132.9609	
Methamidophos-d6	3.75	[M+H]+	124.3	131.0198	97.0242		
Pendimethalin-d5	13.64	[M+H]+	160.2	213.0727	194.0563		

Table S2. LC-HRMS data for the internal standards used for ESI– mode measurements.

Substance	RT [min]	Adduct	CCS [Å²]	Fragment ion 1	Fragment ion 2	Fragment ion 3
Acesulfame-d4	3.61	[M-H]-	124.4	86.0552		
Chloramphenicol-d5	7.97	[M-H]-	166.6	157.0668	262.0654	126.0610
Diclofenac-d4	11.77	[M-H]-	162.8	249.1499	205.1595	193.1008
Diuron-d6	10.27	[M-H]-	147.8	185.9523	149.9755	

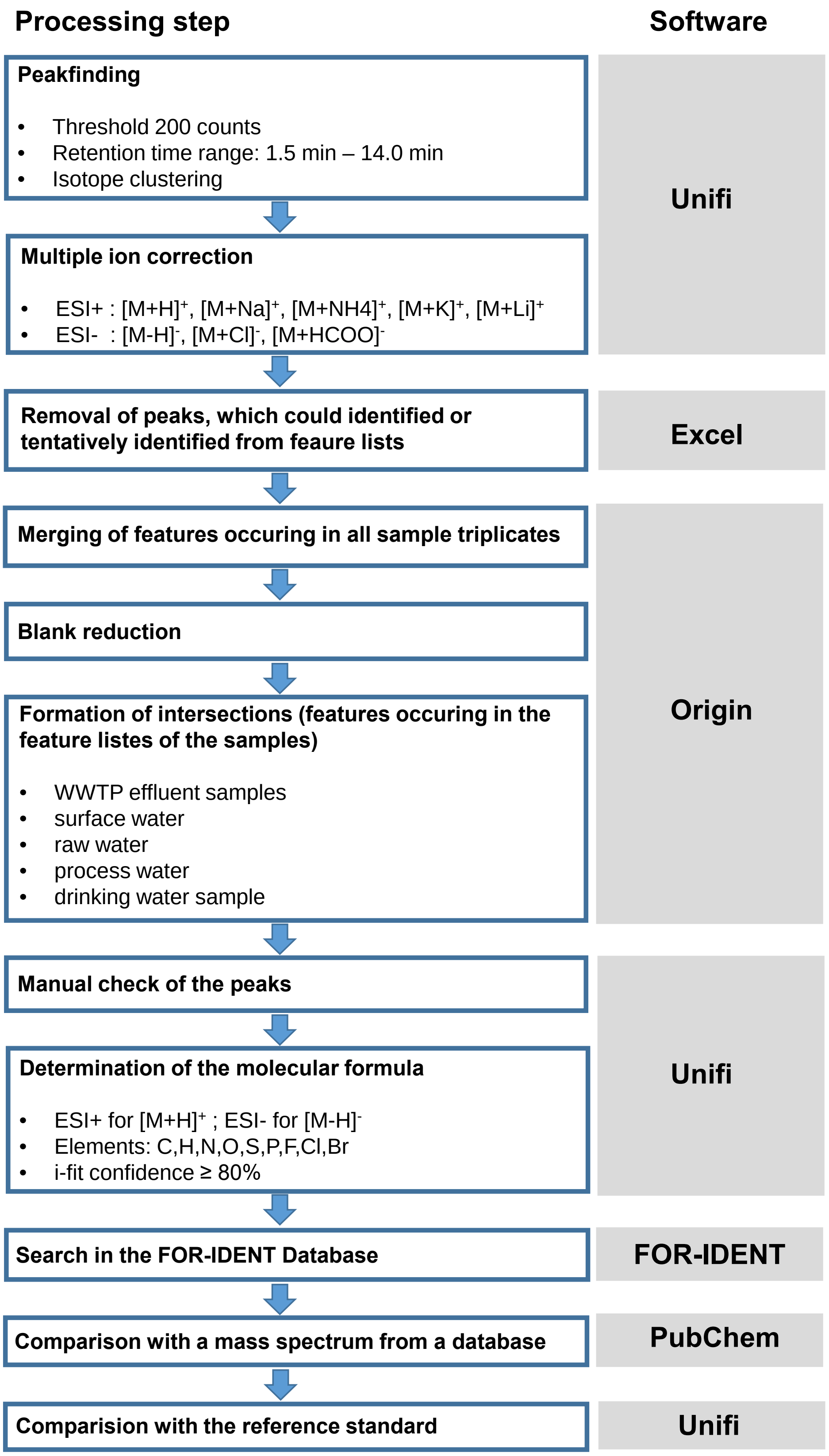

Figure S1. Depiction of the non-target screening analysis workflow.

Table S3. Results from the quantitative screening for each sample. Green marked fields indicate the concentration range < 100 ng/L, yellow marked fields indicate the concentration range between 100-1000 ng/L and red marked fields indicate the concentration range > 1000 ng/L. For not detected compounds n.d. is indicated. For the substances CAS numbers or in case of not available CAS numbers, the InChi Key is stated.

Analytes	CAS number or InChi Key	Drinking water	Process water	Raw water	Surface water	WWTP effluent 1	WWTP effluent 2
1,2-Benzisothiazol-3(2H)-one	2634-33-5	n.d.	n.d.	n.d.	n.d.	n.d.	n.d.
10,11-dihydroxy-10,11-dihydrocarbamazepine	35079-97-1						
1H-Benzotriazole	95-14-7						
2,6-Dichlorbenzamide	2008-58-4	n.d.	n.d.	n.d.	n.d.	n.d.	n.d.
2-Amino-1H-benzimidazole	934-32-7	n.d.	n.d.	n.d.			
4-Acetamidoantipyrine	83-15-8	n.d.					
4-Aminoantipyrine	83-07-8	n.d.	n.d.				
4-chloro-2-methylphenoxyacetic acid (MCPA)	94-74-6	n.d.	n.d.	n.d.	n.d.	n.d.	n.d.
4-Formylaminoantipyrine	1672-58-8	n.d.					
4-Methyl-1H-Benzotriazole/ 5-Methyl-1H-Benzotriazole	29878-31-7 136-85-6	n.d.	n.d.	n.d.			
Acesulfame	55589-62-3	n.d.	n.d.				
Aciclovir	59277-89-3	n.d.	n.d.	n.d.	n.d.		
Aclonifen	74070-46-5	n.d.	n.d.	n.d.	n.d.	n.d.	n.d.
Alachlor	15972-60-8	n.d.	n.d.	n.d.	n.d.	n.d.	n.d.
Aldicarb-sulfone	1646-88-4	n.d.	n.d.	n.d.	n.d.	n.d.	n.d.
Atenolol	29122-68-7	n.d.	n.d.	n.d.			
Atorvastatin	134523-00-5	n.d.	n.d.	n.d.	n.d.	n.d.	n.d.
Atrazine	1912-24-9	n.d.	n.d.	n.d.	n.d.	n.d.	n.d.
Bentazone	25057-89-0	n.d.	n.d.	n.d.	n.d.	n.d.	n.d.
Benzthiazuron	1929-88-0	n.d.	n.d.	n.d.	n.d.	n.d.	n.d.
Betaxolol	63659-18-7	n.d.	n.d.	n.d.	n.d.	n.d.	n.d.
Bezafibrate	41859-67-0	n.d.	n.d.	n.d.	n.d.		n.d.
Bisoprolol	66722-44-9						
Boscalid	188425-85-6	n.d.	n.d.	n.d.	n.d.	n.d.	n.d.
Bromacil	314-40-9	n.d.	n.d.	n.d.	n.d.	n.d.	n.d.
Caffeine	58-08-2	n.d.	n.d.	n.d.	n.d.	n.d.	n.d.
Candesartan	139481-59-7						
Carbamazepine	298-46-4						
Carbetamide	16118-49-3	n.d.	n.d.	n.d.	n.d.	n.d.	n.d.
Carbofuran	1563-66-2	n.d.	n.d.	n.d.	n.d.	n.d.	n.d.
Chloramphenicol	56-75-7	n.d.	n.d.	n.d.	n.d.	n.d.	n.d.
Chloridazon	1698-60-8	n.d.	n.d.	n.d.	n.d.	n.d.	n.d.
Chlortetracycline	57-62-5	n.d.	n.d.	n.d.	n.d.	n.d.	n.d.
Chlorothalonil-M12(*	MTASYHBEZPZGKU-UHFFFAOYSA-M				n.d.	n.d.	n.d.
Chlorothalonil-M5	142733-37-7	n.d.	n.d.	n.d.	n.d.	n.d.	n.d.

Analytes	CAS number or InChi Key	Drinking water	Process water	Raw water	Surface water	WWTP effluent 1	WWTP effluent 2
Chlortoluron	15545-48-9	n.d.	n.d.	n.d.	n.d.	n.d.	n.d.
Ciprofloxacin	85721-33-1	n.d.	n.d.	n.d.	n.d.		n.d.
Clarithromycin	81103-11-9	n.d.					
Desethyl atrazine	6190-65-4	n.d.	n.d.	n.d.	n.d.	n.d.	n.d.
Desethyl terbuthylazine	30125-63-4	n.d.	n.d.	n.d.	n.d.	n.d.	n.d.
Desisopropyl atrazine	1007-28-9				n.d.	n.d.	n.d.
Desphenyl-chloridazon	6339-19-1	n.d.	n.d.	n.d.		n.d.	n.d.
Diatrizoic acid	177-96-4						n.d.
Diclofenac	15307-86-5	n.d.	n.d.	n.d.			
Diethyltoluamide (DEET)	134-62-3						
Diflufenican	83164-33-4	n.d.	n.d.	n.d.	n.d.	n.d.	n.d.
Dimethachlor-CGA369873	1418095-08-5	n.d.	n.d.	n.d.	n.d.	n.d.	n.d.
Dimethenamid	87674-68-8	n.d.	n.d.	n.d.	n.d.	n.d.	n.d.
Dimethenamid-OA	380412-59-9	n.d.	n.d.	n.d.	n.d.	n.d.	n.d.
Dimoxystrobin-505 M08(*	VVBFFEYXSJKVET-HEHNFIMWSA-N	n.d.	n.d.	n.d.	n.d.	n.d.	n.d.
Dimoxystrobin-505 M09(*	RKECPZYSBKSRJM-HEHNFIMWSA-N	n.d.	n.d.	n.d.	n.d.	n.d.	n.d.
Dinoseb	88-85-7	n.d.	n.d.	n.d.	n.d.	n.d.	n.d.
Dinoterb	1420-07-1	n.d.	n.d.	n.d.	n.d.	n.d.	n.d.
Diuron	330-54-1	n.d.	n.d.	n.d.	n.d.	n.d.	n.d.
Epoxiconazole	133855-98-8	n.d.	n.d.	n.d.	n.d.	n.d.	n.d.
Eprosartan	133040-01-4	n.d.	n.d.	n.d.	n.d.		
Ethidimuron	30043-49-3	n.d.	n.d.	n.d.	n.d.	n.d.	n.d.
Flufenacet	142459-58-3	n.d.	n.d.	n.d.	n.d.	n.d.	n.d.
Flufenacet-ESA	947601-87-8	n.d.	n.d.	n.d.	n.d.	n.d.	n.d.
Fluroxypyr	69377-81-7	n.d.	n.d.	n.d.	n.d.	n.d.	n.d.
Flurtamone	96525-23-4	n.d.	n.d.	n.d.	n.d.	n.d.	n.d.
Gabapentin	60142-96-3						
Gabapentin-lactam	64744-50-9						
Glibenclamide	10238-21-8	n.d.	n.d.	n.d.	n.d.	n.d.	n.d.
Hexazinone	51235-04-2	n.d.	n.d.	n.d.	n.d.	n.d.	n.d.
Imidacloprid	138261-41-3	n.d.	n.d.	n.d.	n.d.	n.d.	n.d.
Indometacin	53-86-1	n.d.	n.d.	n.d.	n.d.	n.d.	n.d.
Iodosulfuron-methyl	185119-76-0	n.d.	n.d.	n.d.	n.d.	n.d.	n.d.
Iohexol	66108-95-0	n.d.	n.d.	n.d.		n.d.	n.d.
Iomeprol	78649-41-9	n.d.	n.d.	n.d.		n.d.	
Iopamidol	60166-93-0						n.d.
Iopromide	73334-07-3	n.d.	n.d.	n.d.			
Ioversol	87771-40-2	n.d.	n.d.	n.d.	n.d.	n.d.	n.d.
Ioxithalamin acid	28179-44-4	n.d.	n.d.	n.d.	n.d.	n.d.	n.d.
Ioxynil	1689-83-4	n.d.	n.d.	n.d.	n.d.	n.d.	n.d.
Irbesartan	138402-11-6						

Analytes	CAS number or InChi Key	Drinking water	Process water	Raw water	Surface water	WWTP effluent 1	WWTP effluent 2
Irgarol	28159-98-0	n.d.	n.d.	n.d.	n.d.	n.d.	n.d.
Isoproturon	34123-59-6	n.d.	n.d.	n.d.	n.d.	n.d.	n.d.
Karbutilate	4849-32-5	n.d.	n.d.	n.d.	n.d.	n.d.	n.d.
Ketoprofen	22071-15-4	n.d.	n.d.	n.d.	n.d.		
Lamotrigine	84057-84-1						
Lenacil	2164-08-1	n.d.	n.d.	n.d.	n.d.	n.d.	n.d.
Linuron	330-55-2	n.d.	n.d.	n.d.	n.d.	n.d.	n.d.
Losartan	114798-26-4	n.d.	n.d.	n.d.			
Metalaxyl	57837-19-1	n.d.	n.d.	n.d.	n.d.	n.d.	n.d.
Metalaxyl -CGA108906	104390-56-9	n.d.	n.d.	n.d.	n.d.	n.d.	n.d.
Metalaxyl-CGA62826	87764-37-2	n.d.	n.d.	n.d.	n.d.	n.d.	n.d.
Metamitron	41394-05-2	n.d.	n.d.	n.d.	n.d.	n.d.	n.d.
Metazachlor	67129-08-2	n.d.	n.d.	n.d.	n.d.	n.d.	n.d.
Metazachlor-BH-479-11	1242182-77-9	n.d.	n.d.	n.d.	n.d.	n.d.	n.d.
Metazachlor-BH-479-9	1246215-97-3	n.d.	n.d.	n.d.	n.d.	n.d.	n.d.
Methabenzthiazuron	18691-97-9	n.d.	n.d.	n.d.	n.d.	n.d.	n.d.
Methyl-desphenyl-chloridazon	17254-80-7					n.d.	n.d.
Metolachlor	51218-45-2	n.d.	n.d.	n.d.	n.d.	n.d.	n.d.
Metoprolol	37350-58-6						
Metribuzin	21087-64-9	n.d.	n.d.	n.d.	n.d.	n.d.	
Monolinuron	1746-81-2	n.d.	n.d.	n.d.	n.d.	n.d.	n.d.
N4-Acetylsulfamethoxazole	21312-10-7	n.d.	n.d.	n.d.	n.d.	n.d.	n.d.
Napropamide	15299-99-7	n.d.	n.d.	n.d.	n.d.	n.d.	n.d.
Nicosulfuron	111991-09-4	n.d.	n.d.	n.d.	n.d.	n.d.	
Olmesartan	144689-63-4	n.d.	n.d.	n.d.			
Oxazepam	604-75-1	n.d.	n.d.	n.d.			
Paracetamol	103-90-2	n.d.	n.d.	n.d.	n.d.	n.d.	n.d.
Pentoxifylline	6493-05-6	n.d.	n.d.	n.d.	n.d.	n.d.	n.d.
Phenacetin	62-44-2	n.d.	n.d.	n.d.	n.d.		n.d.
Phenazone	60-80-0	n.d.	n.d.				
Phenobarbital	50-06-6	n.d.	n.d.	n.d.	n.d.	n.d.	n.d.
Picolinafen	137641-05-5			n.d.		n.d.	n.d.
Pindolol	13523-86-9	n.d.	n.d.	n.d.	n.d.	n.d.	n.d.
Propachlor	1918-16-7	n.d.	n.d.		n.d.	n.d.	n.d.
Propiconazole	60207-90-1	n.d.	n.d.	n.d.	n.d.		n.d.
Propranolol	525-66-6	n.d.	n.d.	n.d.			
p-Toluolsulfonamide	70-55-3	n.d.	n.d.	n.d.	n.d.	n.d.	n.d.
Pyraclostrobin	175013-18-0	n.d.	n.d.	n.d.	n.d.	n.d.	n.d.
Quinmerac	90717-03-6	n.d.	n.d.	n.d.	n.d.	n.d.	n.d.
Quinmerac-BH 518-2(*	ZYIDIAPHYHJMCU-UHFFFAOYSA-N	n.d.	n.d.	n.d.	n.d.	n.d.	n.d.
Ritalinic acid	113-45-1				n.d.	n.d.	

**Analytes**	**CAS number or InChi Key**	**Drinking water**	**Process water**	**Raw water**	**Surface water**	**WWTP effluent 1**	**WWTP effluent 2**
Saccharin	81-07-2						
Salicylic acid	69-72-7	n.d.	n.d.	n.d.	n.d.	n.d.	n.d.
Simazine	122-34-9	n.d.	n.d.	n.d.	n.d.	n.d.	n.d.
Simvastatin	79902-63-9	n.d.	n.d.	n.d.	n.d.	n.d.	n.d.
Sitagliptin	486460-32-6						
S-Metolachlor-CGA368208	1173021-76-5	n.d.	n.d.	n.d.	n.d.	n.d.	n.d.
S-Metolachlor-CGA380168	171118-09-5	n.d.			n.d.	n.d.	n.d.
S-Metolachlor-CGA50267	82508-03-0	n.d.	n.d.	n.d.	n.d.	n.d.	n.d.
S-Metolachlor-NOA413173	1418095-19-8	n.d.		n.d.	n.d.	n.d.	n.d.
Sotalol	3930-20-9	n.d.	n.d.	n.d.			n.d.
Sucralose	56038-13-2						
Sulcotrione	99105-77-8	n.d.	n.d.	n.d.	n.d.	n.d.	n.d.
Sulfadiazine	68-35-9	n.d.	n.d.	n.d.	n.d.	n.d.	n.d.
Sulfamethoxazole	723-46-6	n.d.					n.d.
Tebuconazole	107534-96-3	n.d.	n.d.	n.d.	n.d.	n.d.	n.d.
Telmisartan	144701-48-4			n.d.			
Terbutryn	886-50-0	n.d.	n.d.				n.d.
Tetraethylene glycol dimethyl ether	143-24-8	n.d.	n.d.	n.d.	n.d.	n.d.	n.d.
Tolbutamide	64-77-7	n.d.	n.d.	n.d.	n.d.	n.d.	n.d.
Tramadol	27203-92-5	n.d.					
Triadimenol	55219-65-3	n.d.	n.d.	n.d.	n.d.	n.d.	n.d.
Trimethoprim	738-70-5	n.d.	n.d.	n.d.	n.d.		
Tritosulfuron-BH635-4(*	WBSGOTBBRAWAJI-UHFFFAOYSA-N	n.d.	n.d.	n.d.	n.d.	n.d.	n.d.
Valsartan	137862-53-4	n.d.					
Valsartan acid	164265-78-5						
Venlaflaxine	93413-69-5	n.d.					

(* Substances for which CAS numbers were not available. Therefore, the InChi Key is given instead.

Table S4. Outcome of the suspect screening, including substance name, classification, the database which revealed the match, thereby 1 means the database obtained from Wode et al. [1], 2 means the pesticide database from Waters and 3 means the in-house data base. Indent. status means the identification status, whereby i means identified and t means tentatively identified. The *m/z* value, retention time and CCS value were obtained from the first triplicate of the WWTP effluent 1 sample.

**Substance**	**Classification**	**Data base**	**Ident. Status**	***m/z***	**RT [min]**	**CCS [Å²]**	**Response value Drinking water**	**Response value Process water**	**Response value Raw water**	**Response value Surface water**	**Response value WWTP efluent 1**	**Response value WWTP effluent 2**
Flecainide	pharmaceutical	1	i	415.1454	8.9	192.4	545	-	781	9645	64314	166304
Tiapride	pharmaceutical	1	t	329.1533	5.39	183.4	-	98	104	1166	24888	-
Amisulpride	pharmaceutical	1	i	370.1795	6.24	192.5	-	-	-	513	22320	9888
Clindamycin	pharmaceutical	1	i	425.187	8.93	201.9	-	-	-	1983	2111	5352
Fenoterol	pharmaceutical	1	t	304.1554	5.35	168.8	-	-	-	280	2124	3016
Fexofenadine	pharmaceutical	1	i	502.295	9.86	226.5	-	-	-	5553	40634	83680
Melamine	industrial chemical	1,3	i	127.0729	1.23	124.2	-	-	-	3625	3625	5736
Oxcarbazepine	pharmaceutical	1	t	253.0971	9.14	154.6	-	-	-	1056	5600	9448
Sulfapyridine	pharmaceutical	1	t	250.0654	5.74	152.9	-	-	-	666	5355	-
Sulpiride	pharmaceutical	1	i	342.149	4.98	184.7	-	-	-	1767	19453	9075
Tapentadol	pharmaceutical	1	t	222.1856	7.55	152.2	-	-	-	1330	34845	11172
Torasemide	pharmaceutical	1	t	349.1332	9.1	187.3	-	-	-	1144	7756	8640
Celiprolol	pharmaceutical	1	t	380.2544	7.98	204.7	-	-	-	2764	17573	57666
Alprenolol	pesticide	1	t*	250.18 [*)1]	7.38 [*)1]	166.8 [*)1]	-	-	-	155	-	1274
2-(Methylthio)benzothiazole	industrial chemical	1	t	182.009	11.23	132.6	-	-	-	-	439	339
Amitriptyline	pharmaceutical	1	t	278.1903	10.02	166.8	-	-	-	-	15567	21841
Bisnortilidine	metabolite of tilidine	1	t	246.1489	7.96	156	-	-	-	-	612	545
Carbaryl	pesticide	1	t	202.0867 [*)1]	7.09 [*)1]	142.7 [*)1]	-	-	-	-	-	-
Cetirizine	pharmaceutical	1	t	389.1624	10.36	200.3	-	-	-	-	3774	3569
Citalopram	pharmaceutical	1	t	325.1711	8.82	180.1	-	-	-	-	27005	41842
Climbazole	pharmaceutical	1	t	293.1054	9.92	170.8	-	-	-	-	3094	2512
Clopidogrel	pharmaceutical	1	t	322.0662	11.59	168.1	-	-	-	-	2923	1842
Doxepine	pharmaceutical	1	t	280.1697	9.06	163.5	-	-	-	-	9893	7109

Substance	Classification	Data base	Ident. Status	*m/z*	RT [min]	CCS [Å²]	Response value Drinking water	Response value Process water	Response value Raw water	Response value Surface water	Response value WWTP efluent 1	Response value WWTP effluent 2
Melperone	pharmaceutical	1	t	264.1757	7.8	163.4	-	-	-	-	4947	1442
Mirtazapine	pharmaceutical	1	t	266.1655	7.25	160.4	-	-	-	-	5053	4051
Nalidixic acid	pharmaceutical	1	t	233.0915 *)1	8.2 *)1	149.8 *)1	-	-	-	-	-	2166
Nevirapine	pharmaceutical	1	t	267.1243 *)1	8.57 *)1	157.0 *)1	-	-	-	-	-	3455
Pyroquilon	pesticide	1,2	t	174.0915	4.85	137.7	-	-	-	-	23148	19544
Riboflavin	Vitamin B2	1	t	377.1458	7.24	186.2	-	-	-	-	3371	3038
Tilidine	pharmaceutical	1	t	274.1805	7.67	162.1	-	-	-	285	2535	2705
Triamterene	pharmaceutical	1	t	254.1148	7.04	156.4	-	-	-	-	8760	11009
Verapamil	pharmaceutical	1	i	455.2903	9.43	210.9	-	-	-	-	3169	1444
Mexacarbate	pesticide	1,2	t	223.1434 *)1	7.26 *)1	155.5 *)1	-	-	-	-	-	544
Thiabendazole	pesticide	1,2	t	202.0447	6.71	139	-	-	-	-	818	466
2-Ethylidene-1,5-dimethyl-3,3 -diphenylpyrrolidine (EDDP)	metabolite of methadone	1	t	278.1904	8.92	165.8	-	-	-	-	10424	15383
Ketamine	pharmaceutical	1	t	238.0988	7.02	149.6	-	-	-	-	1469	4338
Metoclopramide	pharmaceutical	1	t	300.1477	6.77	172.2	-	-	-	-	2122	-
Propafenone	pharmaceutical	1	t	342.2063	9.83	177.3	-	-	-	-	22554	34360
Ranitidine	pharmaceutical	1	t	315.1483	5.08	168.6	-	-	-	-	5207	11336
Zolpidem	pharmaceutical	1	t	308.176	7.84	177.5	-	-	-	-	3288	-
Ethoxyquin	pesticide	2	t	218.1545	7.39	153.8	-	-	-	-	264	-
Anhydroerythromycin	pharmaceutical	3	i	716.4565	10.29	269.8	-	-	-	-	432	-

*For alprenolol, the CCS value does not match with the CCS value from the literature (see Table S5).
*)1 *m/z*, retention time and CCS value were obtained from the first triplicate of the WWTP effluent 2 sample.
*)2 *m/z*, retention time and CCS value were obtained from the first triplicate of the surface water sample.

Table S5. Comparison of measured CCS values with reference CCS (determined with the reference standard) and CCS values from literature. The CCS error was calculated as $\frac{CCS_{measured}-CCS^*}{CCS_{measured}}$ [2], thereby CCS* means the CCS value of the reference standard or the CCS value obtained from literature.

Substance	PubChem ID	CCS sample	CCS ref. Std.	CCS error	CCS pesticides library	CCS error	CCS literature	CCS error
		[Å²]	[Å²]	[%]	[Å²]	[%]	[Å²]	[%]
Flecainide	3356	192.4	193.4	-0.52			192.2 *(b	0.10
Amisulpride	2159	192.5	194.1	-0.83			193.1 *(b	-0.31
Clindamycin	446598	201.9	203.3	-0.69			202.5 *(a	-0.29
Fenoterol	3343	168.8					171.1 *(a	-1.34
Fexofenadine	3348	226.5	227.2	-0.31			226.9 *(b	-0.18
Melamine	7955	124.2	123.3	0.72				
Oxcarbazepine	34312	154.6					153.8 *(b	0.52
Sulfapyridine	5336	152.9					152.7 *(a<br>150.8 *(b<br>154.6 *(c	0.13<br>1.37<br>-1.11
Sulpiride	5355	184.7	186.4	-0.92			184.3 *(b	0.22
Tapentadol	9838022	152.2					152.1 *(b	0.07
Alprenolol	2119	166.8					159.5 *(b	4.38
Amitriptyline	2160	166.8					167.2 *(b	-0.24
Cetirizine	2678	200.3					200.8 *(b	-0.25
Citalopram	2771	180.1					180.1 *(b	0.00
Clopidogrel	60606	168.1					168.7 *(a	-0.33
Doxepine	3158	163.5					164.3 *(b	-0.49
Melperone	15387	163.4					163.5 *(b	-0.06
Mirtazapine	4205	160.4					161.0 *(b	-0.37
Nalidixic acid	4421	149.8					145.8 *(a<br>148.0 *(c	2.70<br>1.20
Pyroquilon	91665	137.7			136.0	1.23		
Verapamil	2520	210.9	208.8	1.00			210.2 *(a	0.33
Mexacarbate	9414	155.5			155.6	-0.04		
Thiabendazole	5430	139.0			138.2	0.57	137.4 *(a<br>141.7 *(c	1.12<br>-1.94
Ketamine	3821	149.6					148.8 *(a<br>149.2 *(b	0.51<br>0.27
Metoclopramide	4168	172.2					173.1 *(a<br>173.2 *(b	-0.51<br>-0.58
Propafenone	4932	177.3					178.5 *(b	-0.68
Zolpidem	5732	177.5					177.2 *(b	0.17
Ethoxyquin	3293	153.8			153.8	0.02	154.5 *(a	-0.42
Anhydroerythromycin	71313417	269.8	269.2	0.22				

*a) Reference of CCS value: Celma et al. 2020 [3]
*b) Reference of CCS value: Mollerup et al. 2018 [4]
*c) Reference of CCS value: Tejada-Casado et al. 2018 [5]

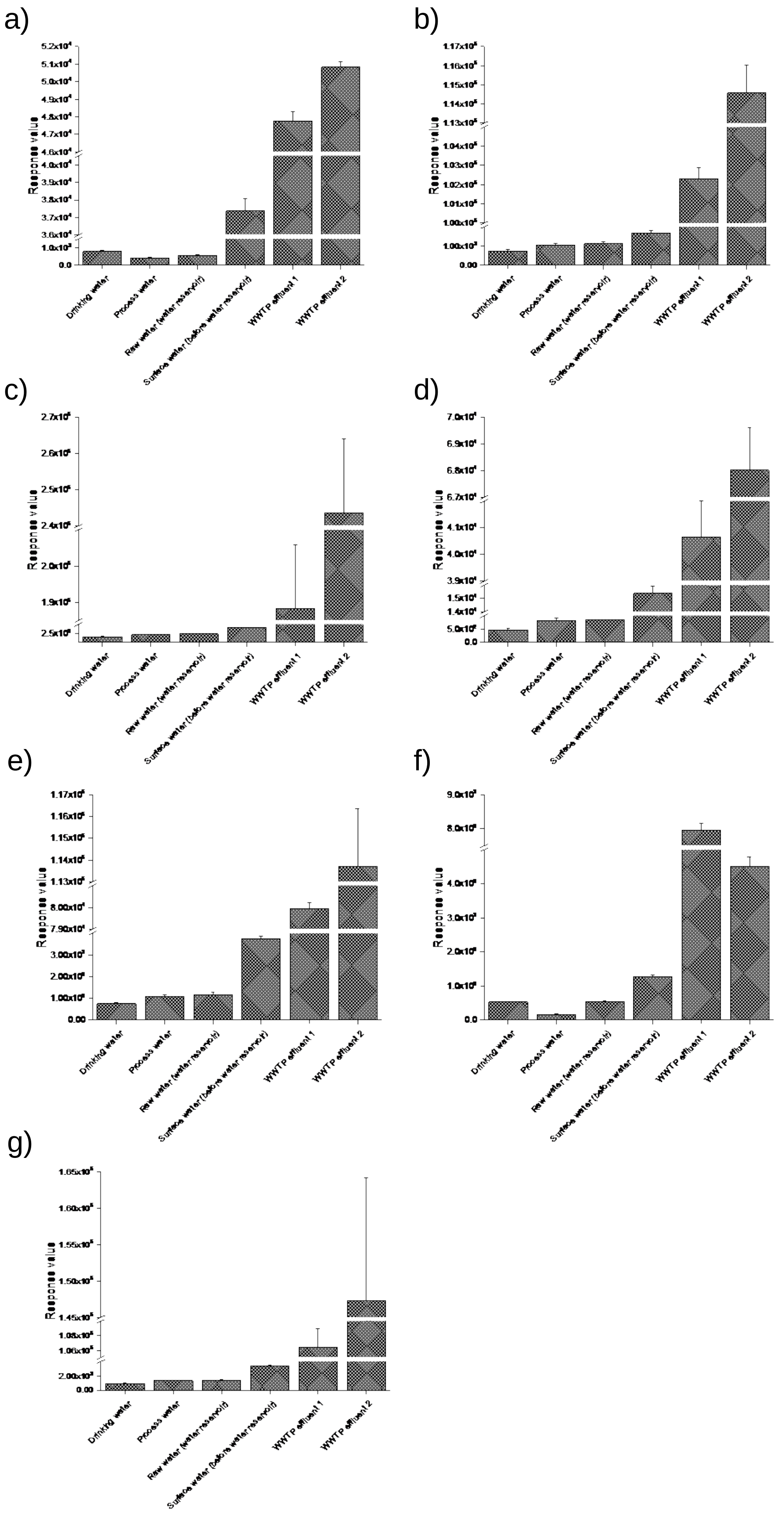

Figure S2. Representation of detected response values for a) feature 1, b) feature 2, c) feature 3, d) feature 4, e) feature 5, f) feature 6 and g) feature 7 in ESI+ mode.

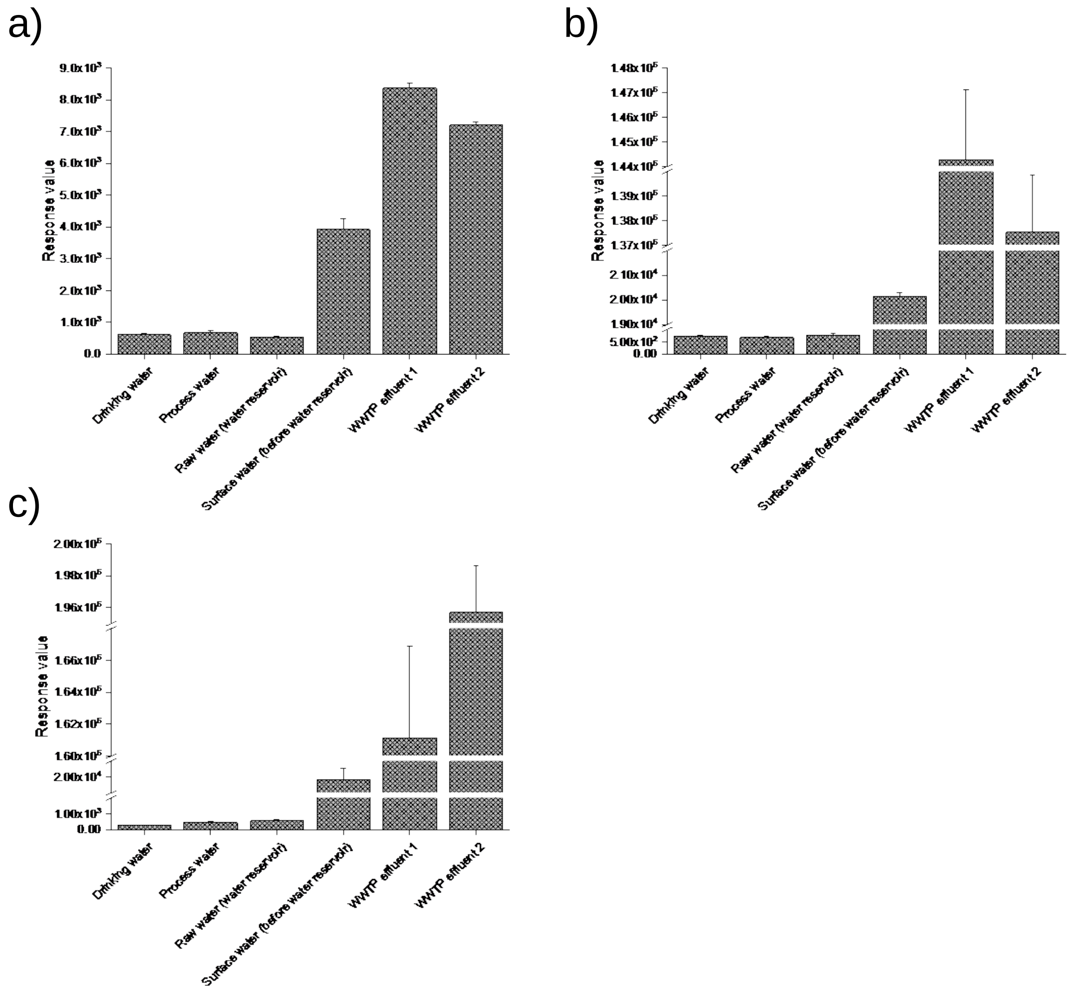

Figure S3. Representation of detected response values for a) feature 1, b) feature 2 and c) feature 3 in ESI- mode.

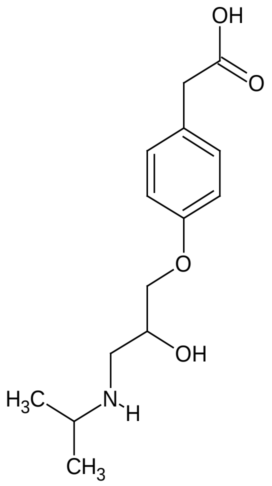

Figure S4. Chemical structure of metoprolol acid/atenolol acid.

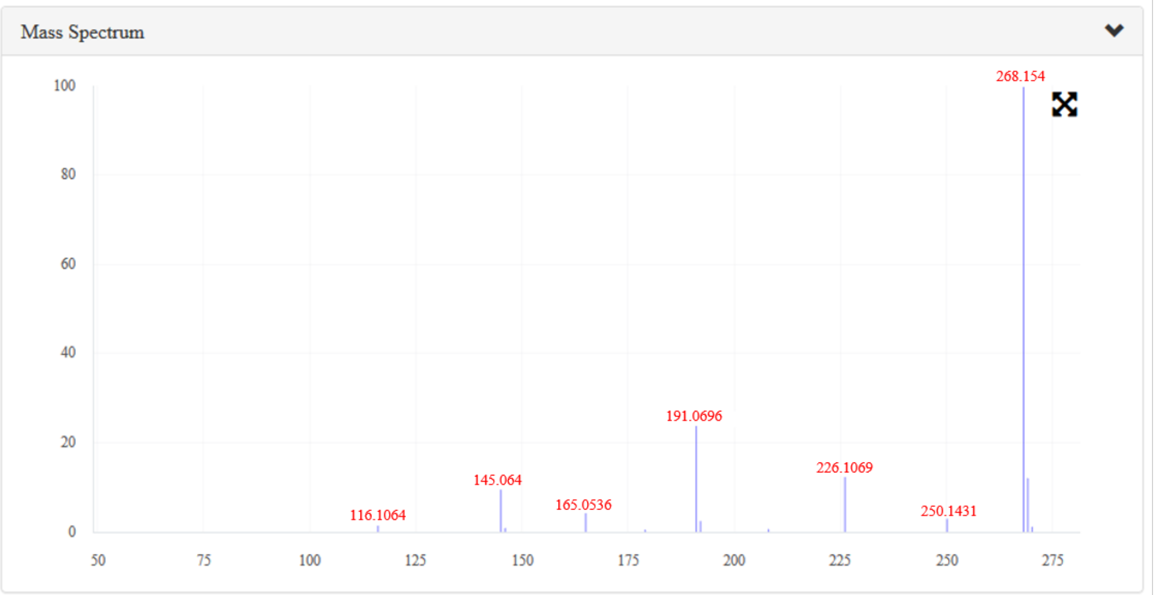

Figure S5. Fragment ion spectrum for metoprolol acid/atenolol acid obtained from PubChem.org [6].

Table S6. Calculations for the Kendrick mass defect plot according to Thurman et al. [7]. For the calculation of the Kendrick mass, the Kendrick mass scaling factor of 0.999 404 559 was calculated (44.0000/44.0262 = 0.999 404 559) and this factor was multiplied with the measured *m/z* values. The nominal Kendrick mass means the Kendrick mass nearest integer value [8]. Afterwards, the Kendrick mass detect was calculated by the subtraction of the nominal Kendrick mass with the Kendrick mass.

m/z	**Molecular formula (all as $[M+H]^+$ ions)**	**Kendrick mass**	**Nominal Kendrick mass**	**Kendrick mass defect**
**247.1153**	$C_9H_{17}F_3O_4$	246.9682	247.0000	0.0318
**291.1416**	$C_{11}H_{21}F_3O_5$	290.9682	291.0000	0.0318
**335.1679**	$C_{13}H_{25}F_3O_6$	334.9684	335.0000	0.0316
**379.1940**	$C_{15}H_{29}F_3O_7$	378.9682	379.0000	0.0318
**423.2203**	$C_{17}H_{33}F_3O_8$	422.9683	423.0000	0.0317
**467.2468**	$C_{19}H_{37}F_3O_9$	466.9685	467.0000	0.0315
**511.2727**	$C_{21}H_{41}F_3O_{10}$	510.9683	511.0000	0.0317

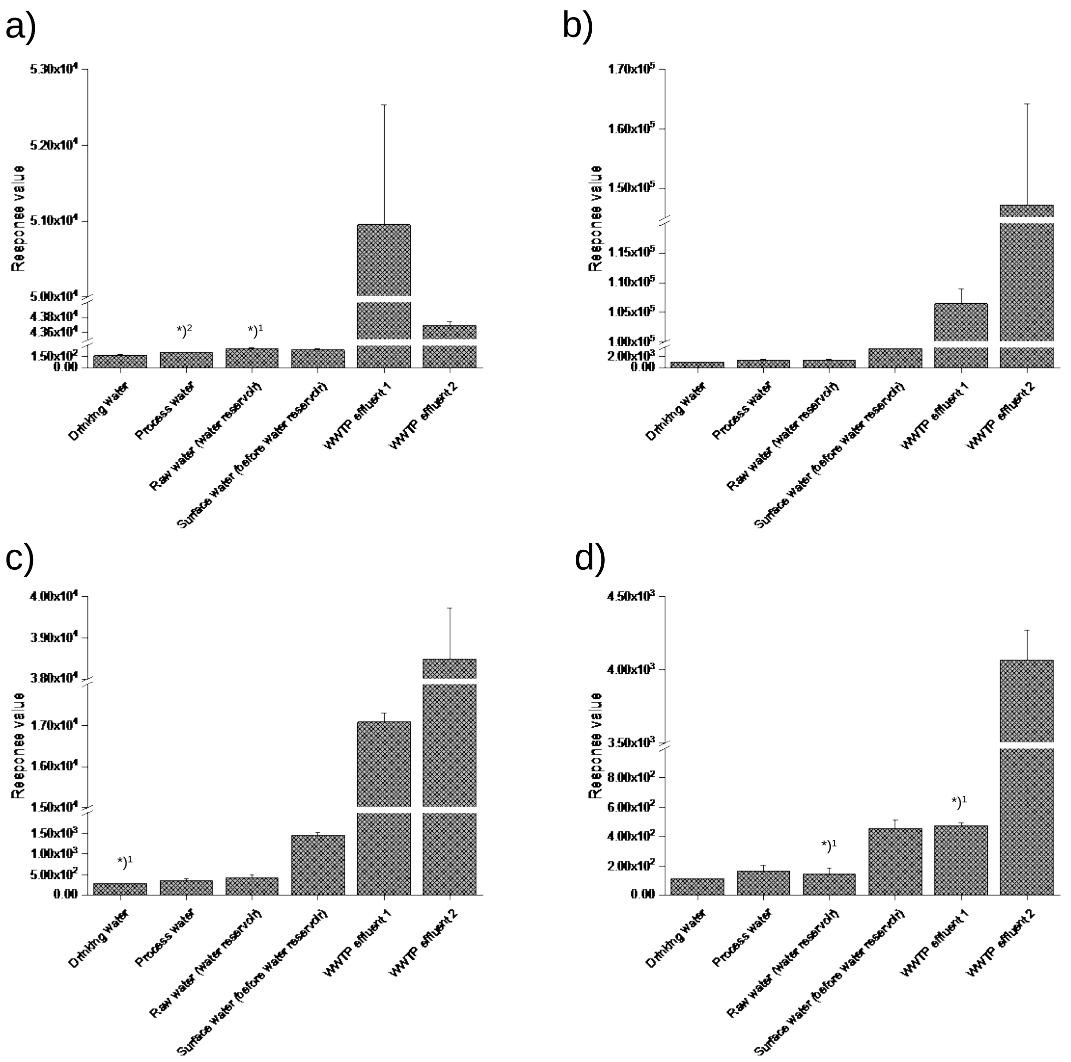

Figure S6. Representation of detected response values for a) *m/z* 247.1153, b) 379.1940, c) 467.2468 and d) 511.2727. *)1 means that only in two of three replicate signals were detected and *)2 means that in only one of three replicates a signal was detected.

Table S7. Molecular formulas for the features, included in the homologous series. The i-fit confidence is indicated in brackets.

m/z	Molecular formula for the $[M+Na]^+$ adduct
247.1153	C10H23FP2 (83%)
291.1416	C11H24O7 (70%)<br>C12H27FOP2 (24%)
335.1679	C13H28O8 (100%)
379.1940	C15H32O9 (50%)<br>C15H31F2N2O3 (22%)<br>C16H35FO3P2 (14%)

m/z	**Molecular formula for the $[M+Na]^+$ adduct**
423.2203	C17H36O10 (31%)<br>C18H39FO4P2 (18%)<br>C17H35F2N2O4P (16%)<br>C12H33N8O5P (13%)
467.2468	C14H37N8O6P (91%)
511.2727	C24H42F5O2P (15%)<br>C9H33FN20OS (11%)

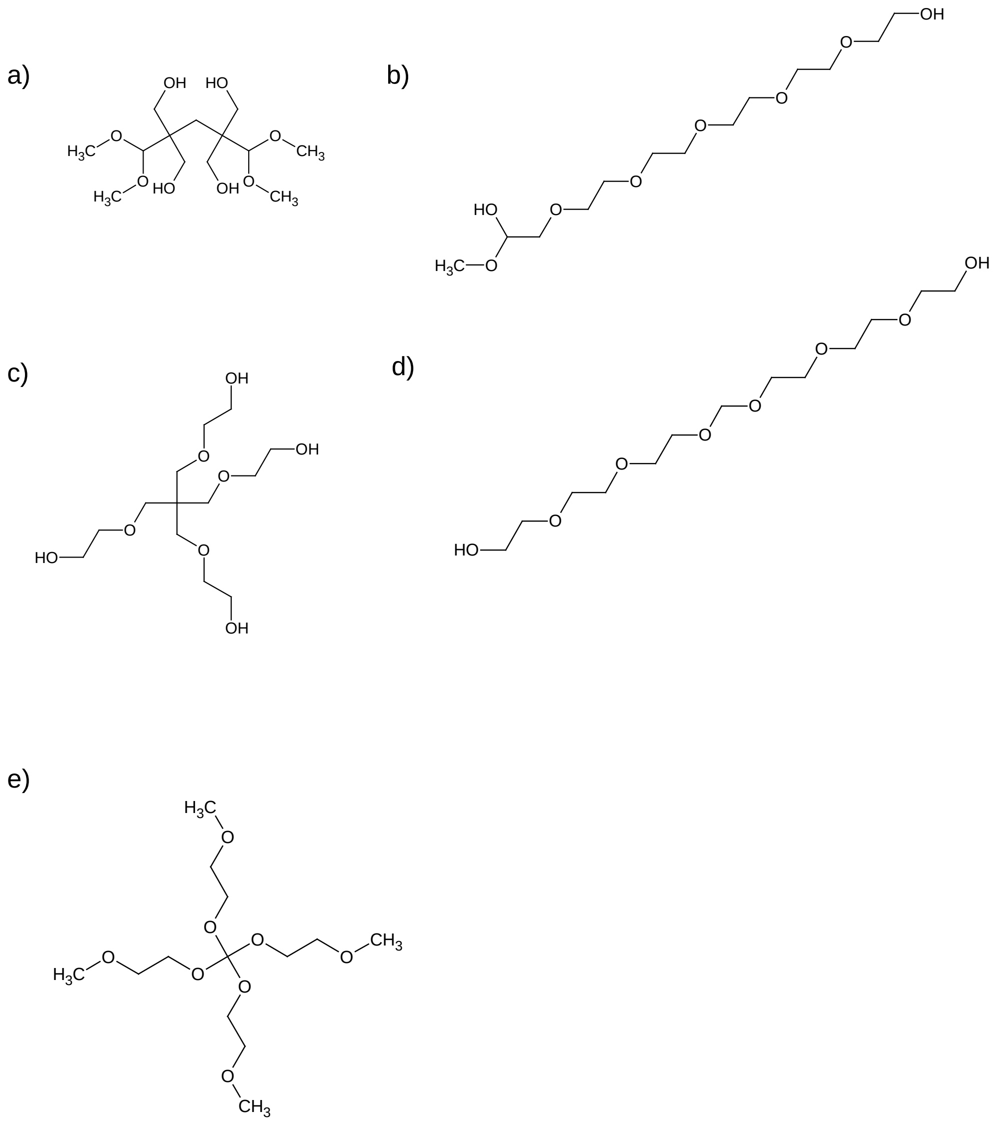

Figure S7. Chemical structures of a) 2,4-Bis(dimethoxymethyl)-2,4-bis(hydroxymethyl)-1,5-pentanediol, b) 2,5,8,11,14,17-Hexaoxanonadecane-3,19-diol, c) 2-{3-(2-Hydroxyethoxy)-2,2-bis[(2-hydroxyethoxy)methyl]propoxy}ethanol, d) 3,6,9,11,14,17-Hexaoxanonadecane-1,19-diol and e) 6,6-Bis(2-methoxyethoxy)-2,5,7,10-tetraoxaundecane.

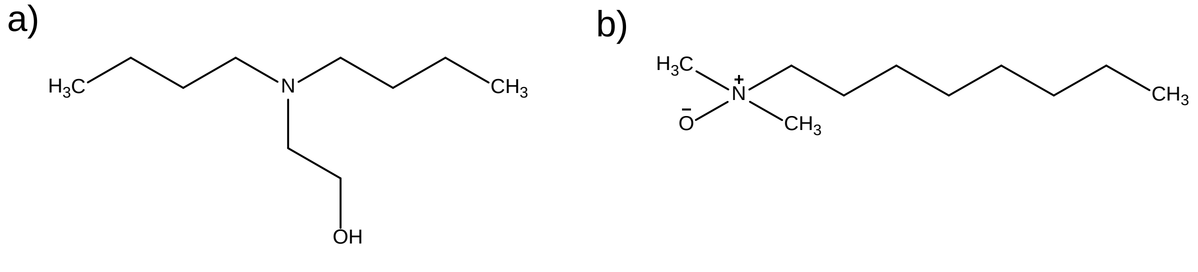

Figure S8. Chemical structures of a) N,N-Dibutylethanolamine and b) N,N-Dimethyloctylamine-N-oxide.

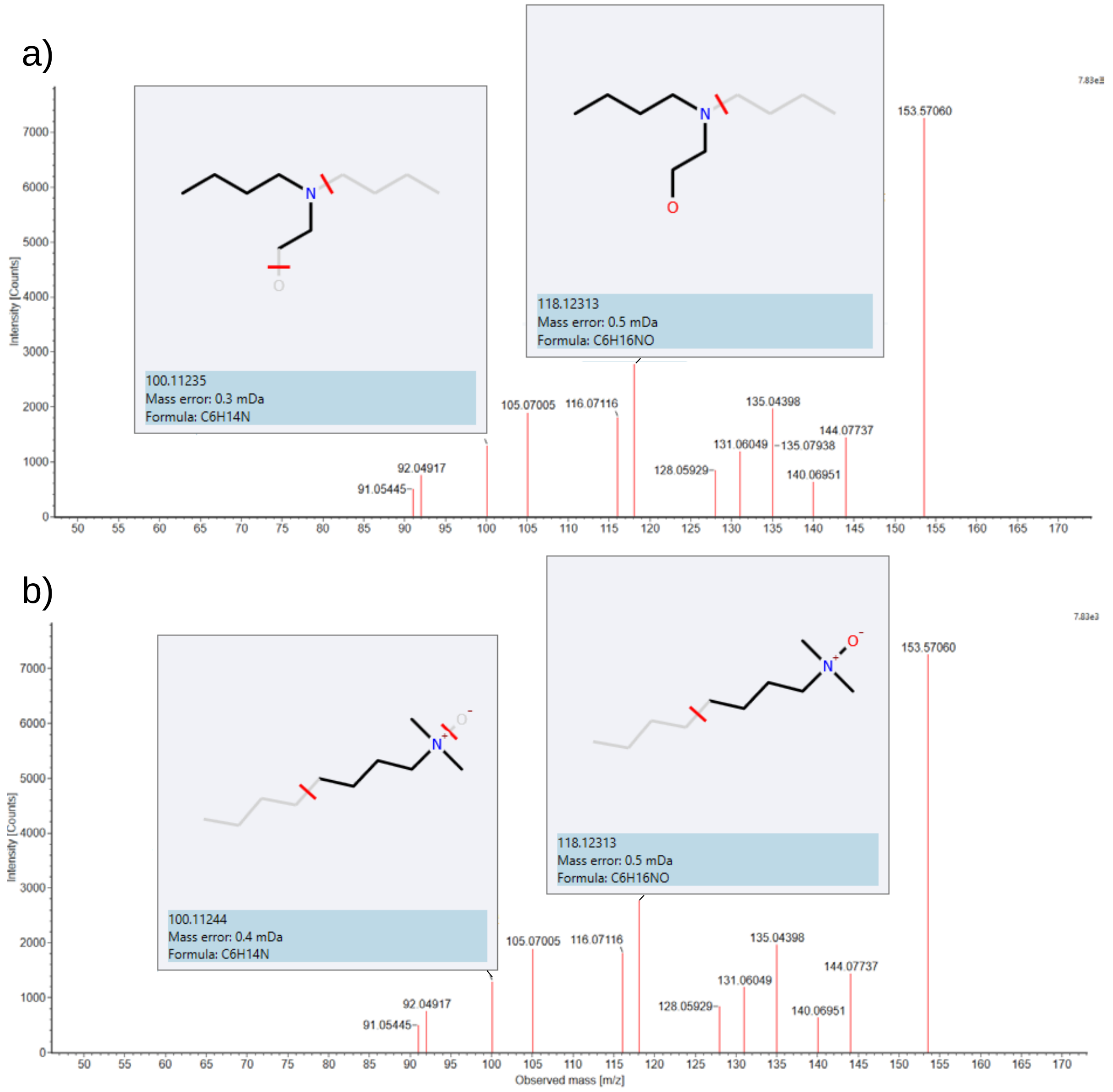

Figure S9. Comparison of measured fragment ion spectra for feature 6 in WWTP effluent 1 with in-silico fragmentation of a) N,N-Dibutylethanolamine and b) N,N-Dimethyloctylamine-N-oxide.

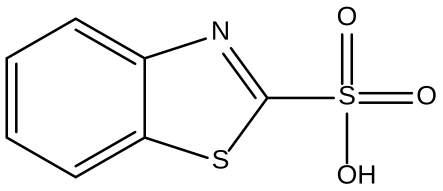

Figure S10. Chemical structure of 1,3-benzothiazole-2-sulfonic acid.

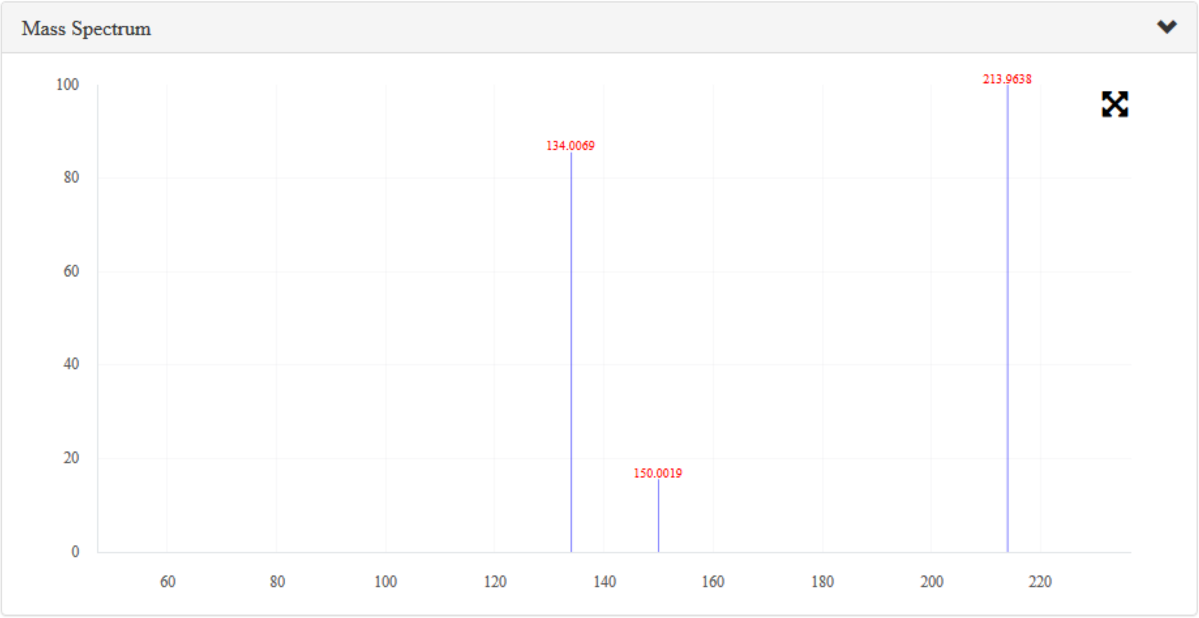

Figure S11. Fragment ion spectrum obtained by PubChem.org [9] for 1,3-benzothiazole-2-sulfonic acid.

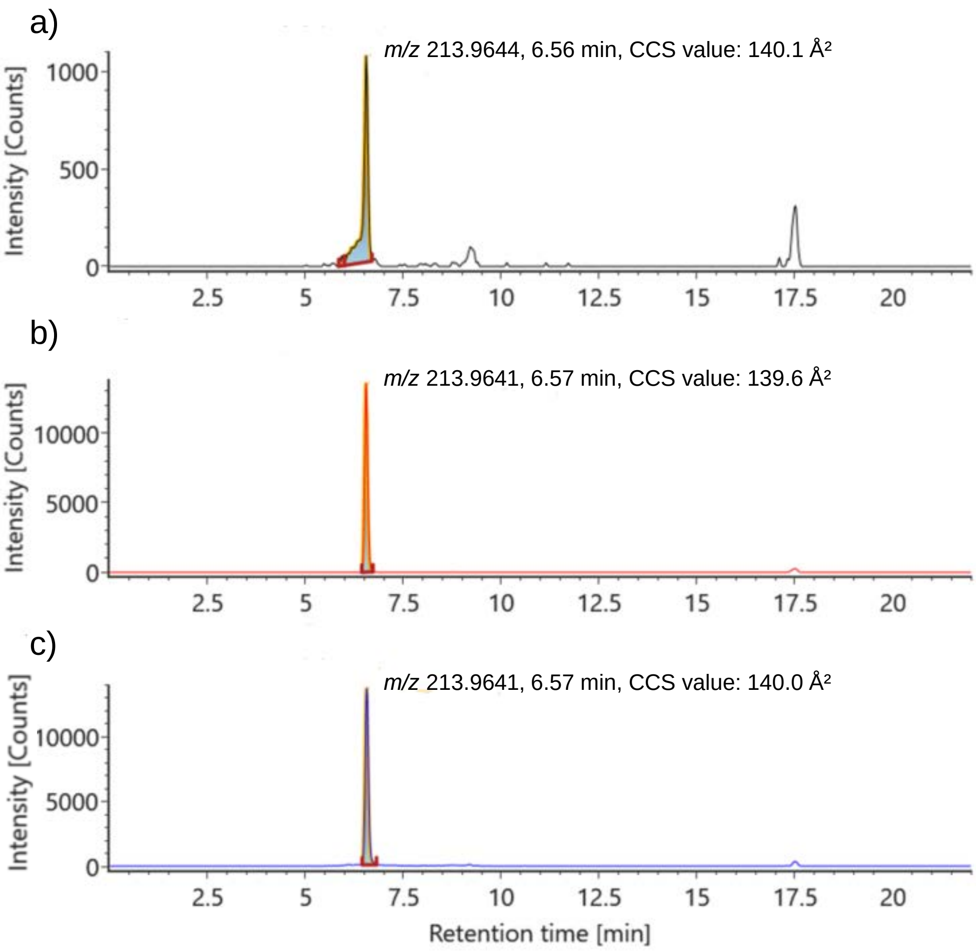

Figure S12. Extracted ion chromatograms of 1,3-benzothiazole-2-sulfonic acid for a) the drinking water sample, b) the WWTP effluent 2 sample and c) for the reference standard (500 ng/L) in a 30 ppm mass window.

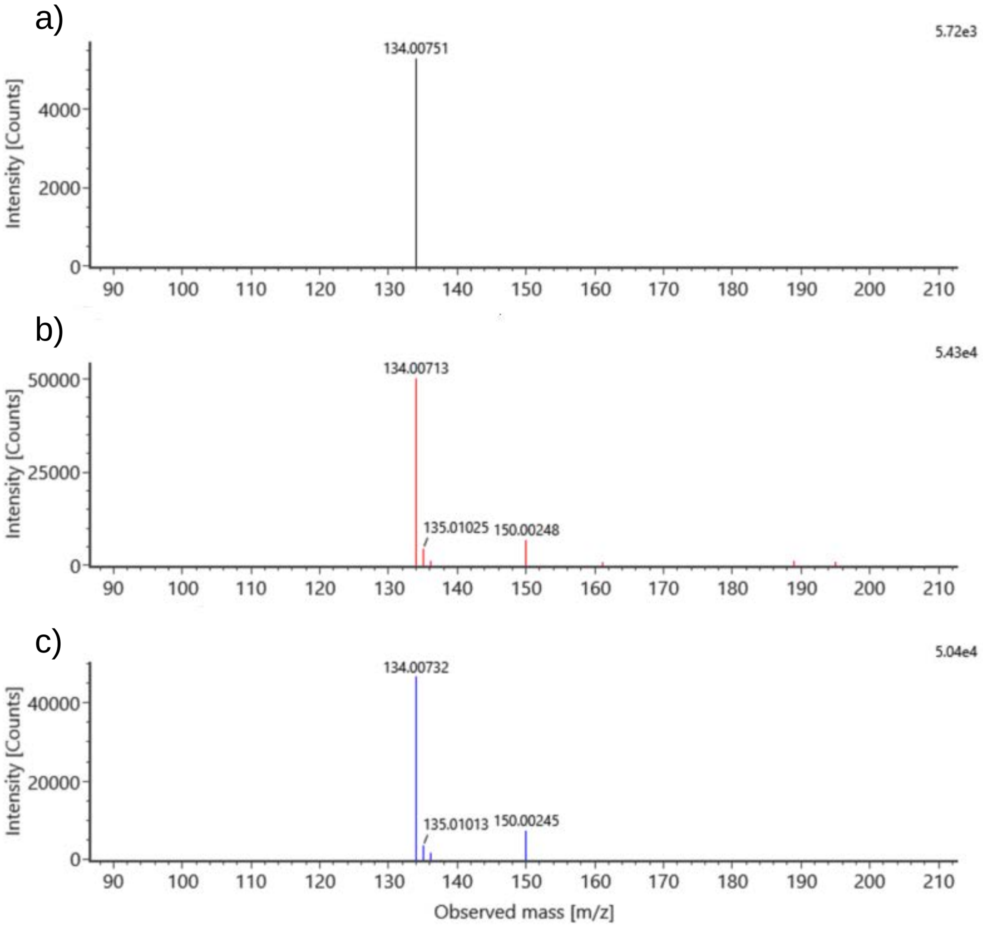

Figure S13. Fragment ion spectra of 1,3-benzothiazole-2-sulfonic acid for a) the drinking water sample, b) the WWTP effluent 2 sample and c) for the reference standard (500 ng/L). Spectra were recorded by a collision energy ramp from 15 eV to 40 eV from the precursor *m/z* 213.9643 using the HDMSe scan mode.

References

[1] Wode F, van Baar P, Dünnbier U, Hecht F, Taute T, Jekel M, Reemtsma T. Search for over 2000 current and legacy micropollutants on a wastewater infiltration site with a UPLC-high resolution MS target screening method. Water Res. 2015;69:274-283.

[2] Goscinny S, McCullagh M, Far J, De Pauw E, Eppe G. Towards the use of ion mobility mass spectrometry derivedcollision cross section as a screening approach for unambiguous identification of targeted pesticides in food. Rapid Commun. Mass Spectrom. 2019;33:34-48.

[3] Celma A, Sancho JV, Schymanski EL, Fabregat-Safont D, Ibáñez M, Goshawk J, Barknowitz G, Hernández F, Bijlsma L. Improving target and suspect screening high-

resolution mass spectrometry workflows in environmental analysis by ion mobility separation. Environ. Sci. Technol. 2020;54:15120-15131.
[4] Mollerup CB, Mardal M, Dalsgaard PW, Linnet K, Barron LP. Prediction of collision cross section and retention time for broad scope screening in gradient reversed-phase liquid chromatography-ion mobility-high resolution accurate mass spectrometry. J. Chromatogr. A 2018;1542: 82-88.
[5] Tejada-Casado C, Hernández-Mesa M, Monteau F, Lara FJ, del Olmo-Iruela M, García-Campaña AM, Le Bizec B, Dervilly-Pinela G. Collision cross section (CCS) as a complementary parameter to characterize human and veterinary drugs. Anal. Chim. Acta 2018;1043:52-63.
[6] PubChem database: https://pubchem.ncbi.nlm.nih.gov/compound/Metoprolol-acid#section=Mass-Spectrometry
Accessed on February 4, 2019.
[7] Thurman EM, Ferrer I, Blotevogel J, Borch T. Analysis of hydraulic fracturing flowback and produced waters using accurate mass: identification of ethoxylated surfactants. Anal. Chem. 2014;86:9653-9661.
[8] Gross J. Massenspektrometrie: Ein Lehrbuch. First edition, Springer-Verlag, Berlin, Heidelberg, 2013, p. 116.
[9] PubChem database: https://pubchem.ncbi.nlm.nih.gov/compound/Benzothiazole-2-sulfonic-acid
Accessed on February 4, 2019
